# Supplementary figures and images for: Integration of molecular networking and fingerprint analysis for studying constituents in Microctis Folium
Source: PLoS One. 2020 Jul 7;15(7):e0235533. doi: 10.1371/journal.pone.0235533 (PMC7340309; doi:10.1371/journal.pone.0235533)

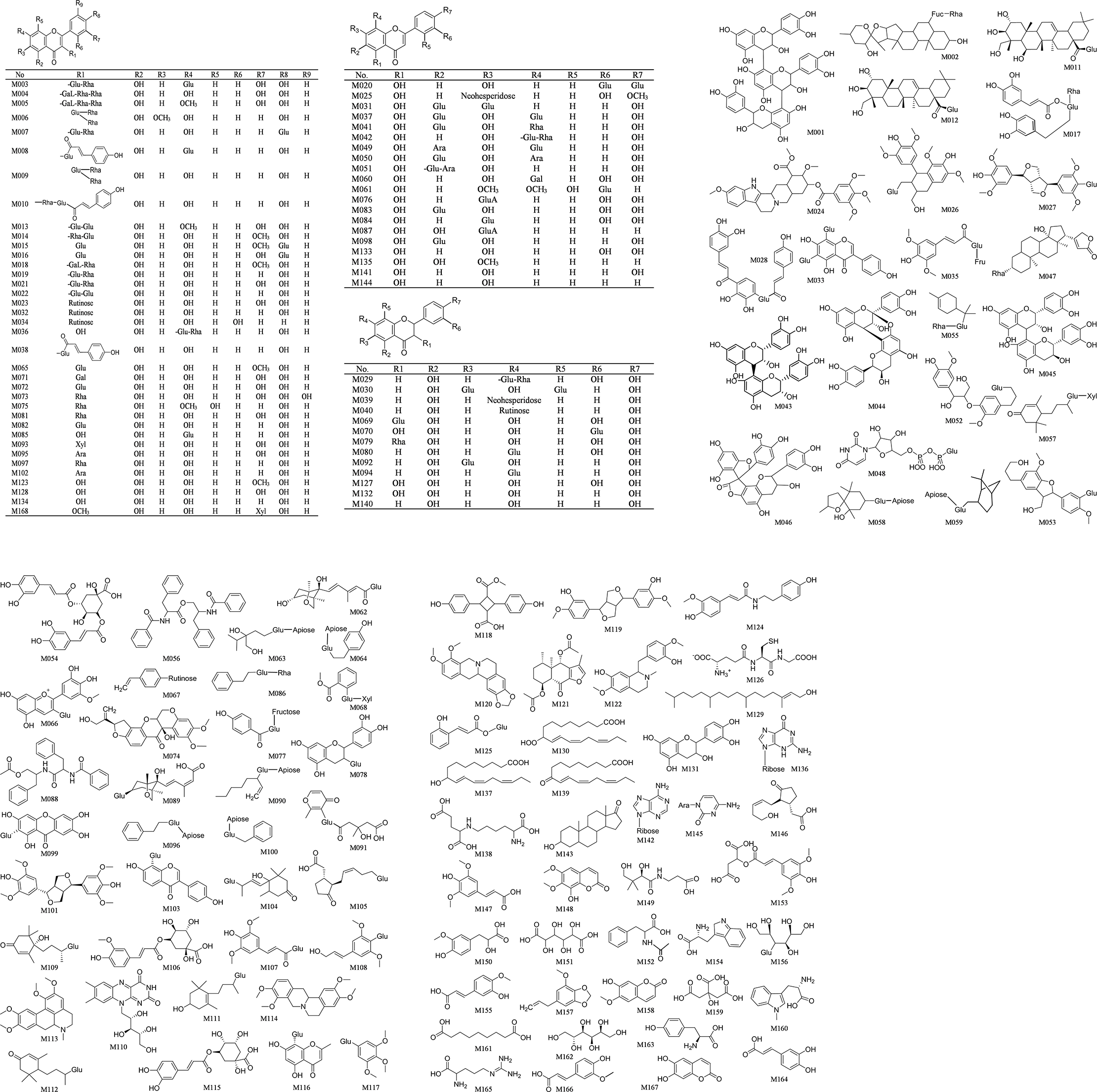

Supplement: S1 Fig — (TIF) [file pone.0235533.s001.tif]

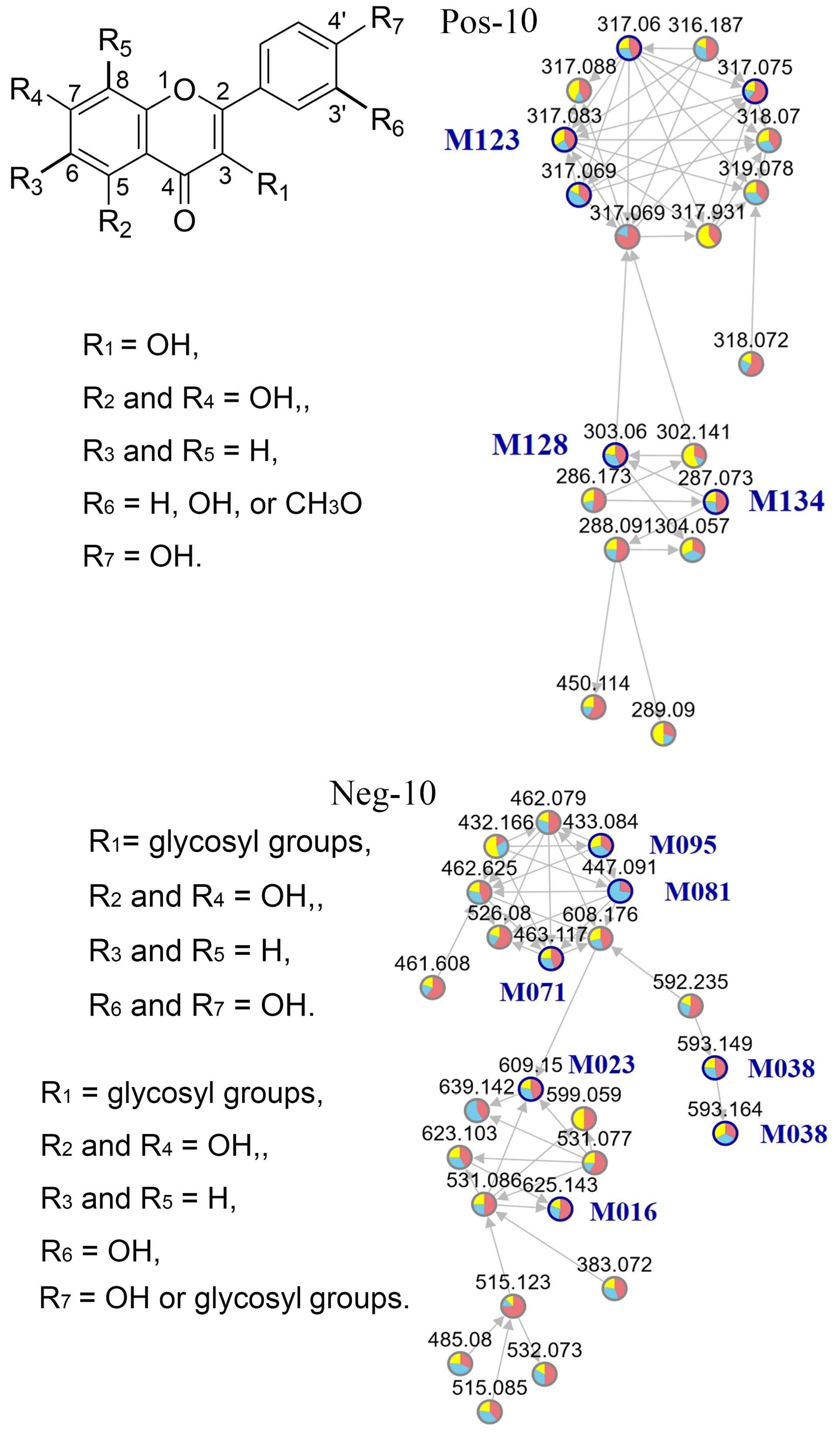

Supplement: S2 Fig — Each node is represented as a compound’s MS/MS spectrum because all retention times of nodes are different. Blue nodes are represented as spectra of identified constituents, which include noted compounds’ names, and grey nodes are represented as spectra of their unknown analogues. Each coloured wedge of a node pie is the proportion of the spectral counts derived from each respective season (red: summer, blue: autumn, yellow: winner). Each edge between nodes is represented as a spectrum-to-spectrum similarity cosine score above 0.7. (TIF) [file pone.0235533.s002.tif]

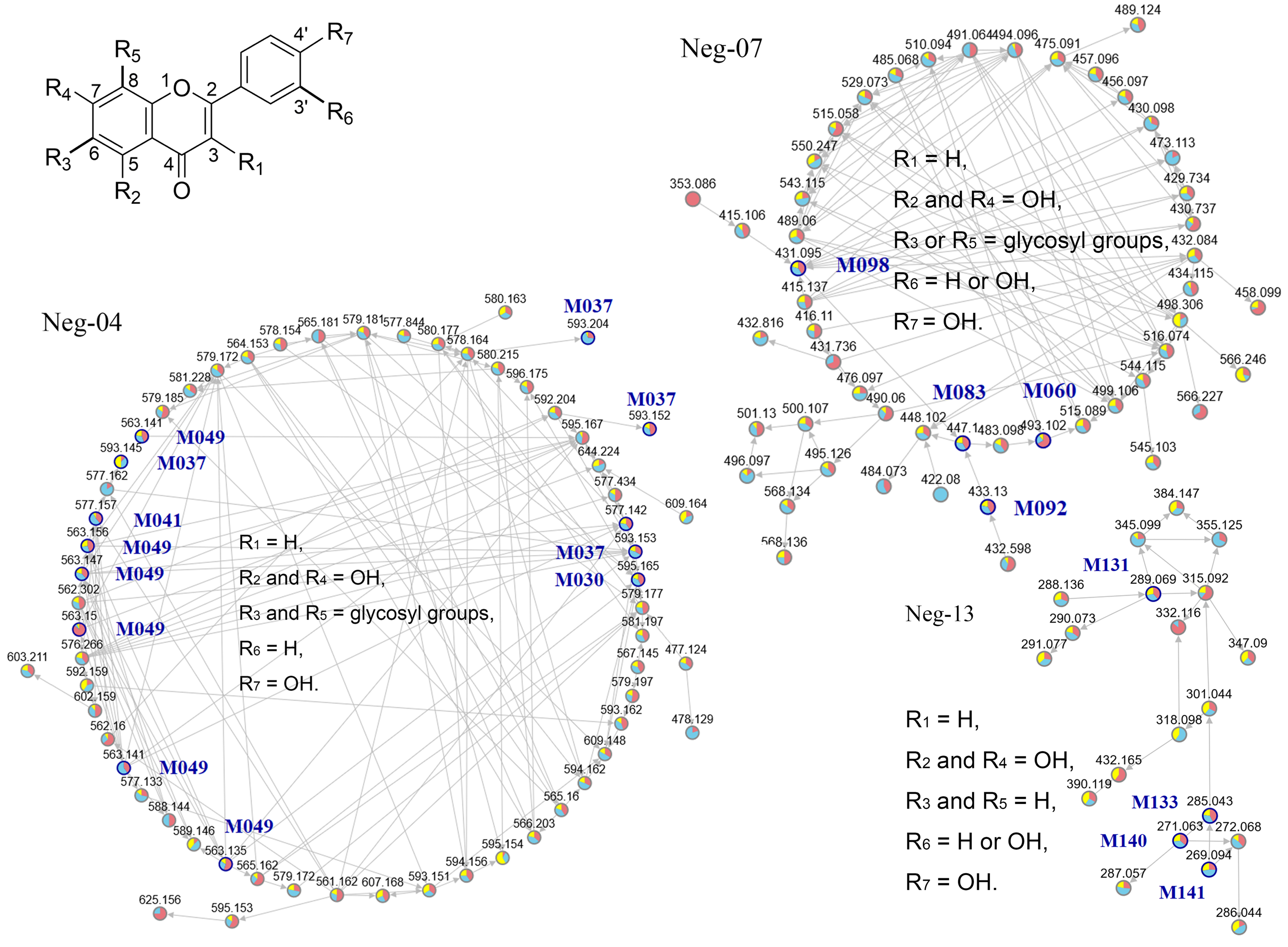

Supplement: S3 Fig — Each node is represented as a compound’s MS/MS spectrum because all retention times of nodes are different. Blue nodes are represented as spectra of identified constituents, which include noted compounds’ names, and grey nodes are represented as spectra of their unknown analogues. Each coloured wedge of a node pie is the proportion of the spectral counts derived from each respective season (red: summer, blue: autumn, yellow: winner). Each edge between nodes is represented as a spectrum-to-spectrum similarity cosine score above 0.7. (TIF) [file pone.0235533.s003.tif]

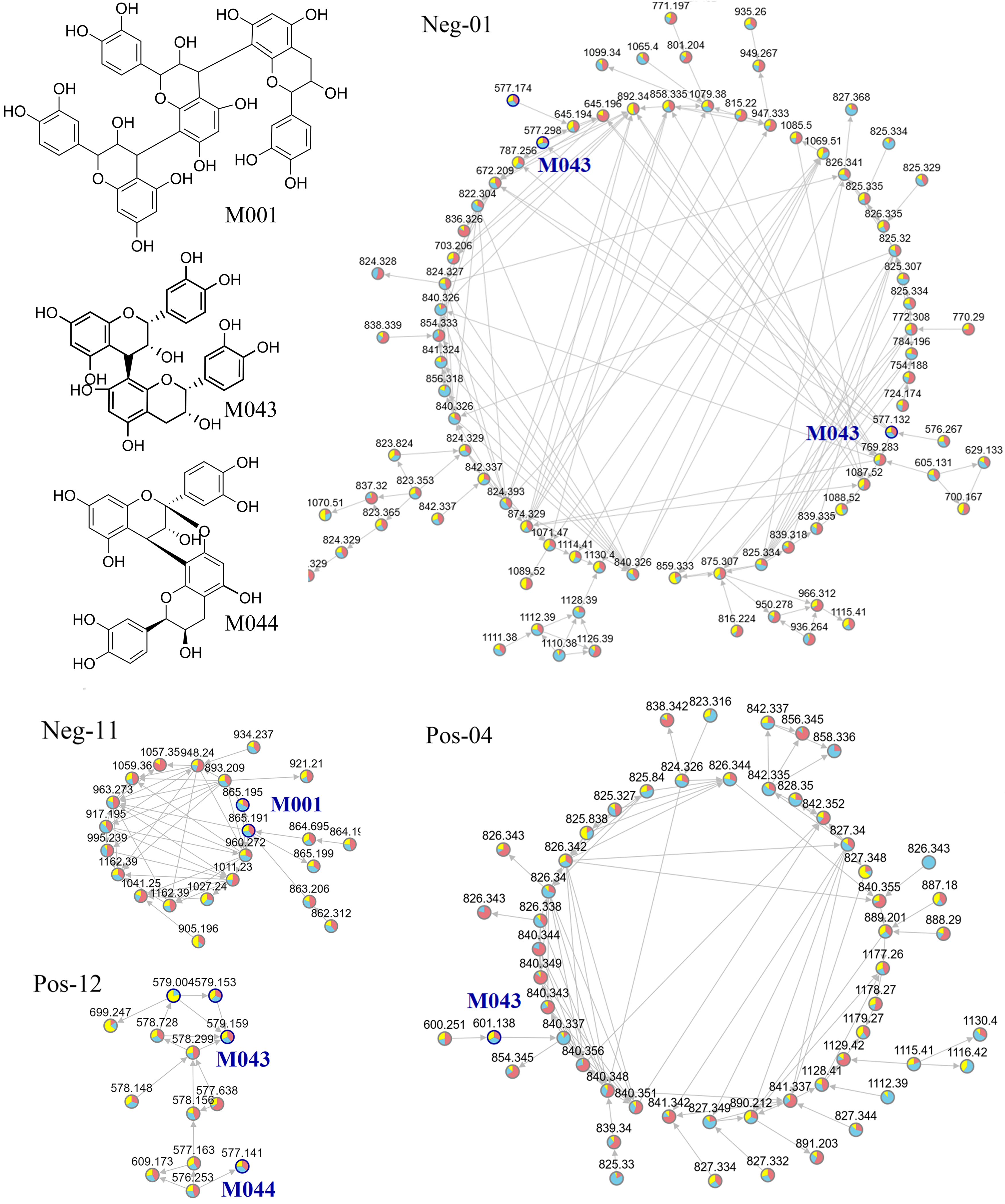

Supplement: S4 Fig — Each node is represented as a compound’s MS/MS spectrum because all retention times of nodes are different. Blue nodes are represented as spectra of identified constituents, which include noted compounds’ names, and grey nodes are represented as spectra of their unknown analogues. Each coloured wedge of a node pie is the proportion of the spectral counts derived from each respective season (red: summer, blue: autumn, yellow: winner). Each edge between nodes is represented as a spectrum-to-spectrum similarity cosine score above 0.7. (TIF) [file pone.0235533.s004.tif]

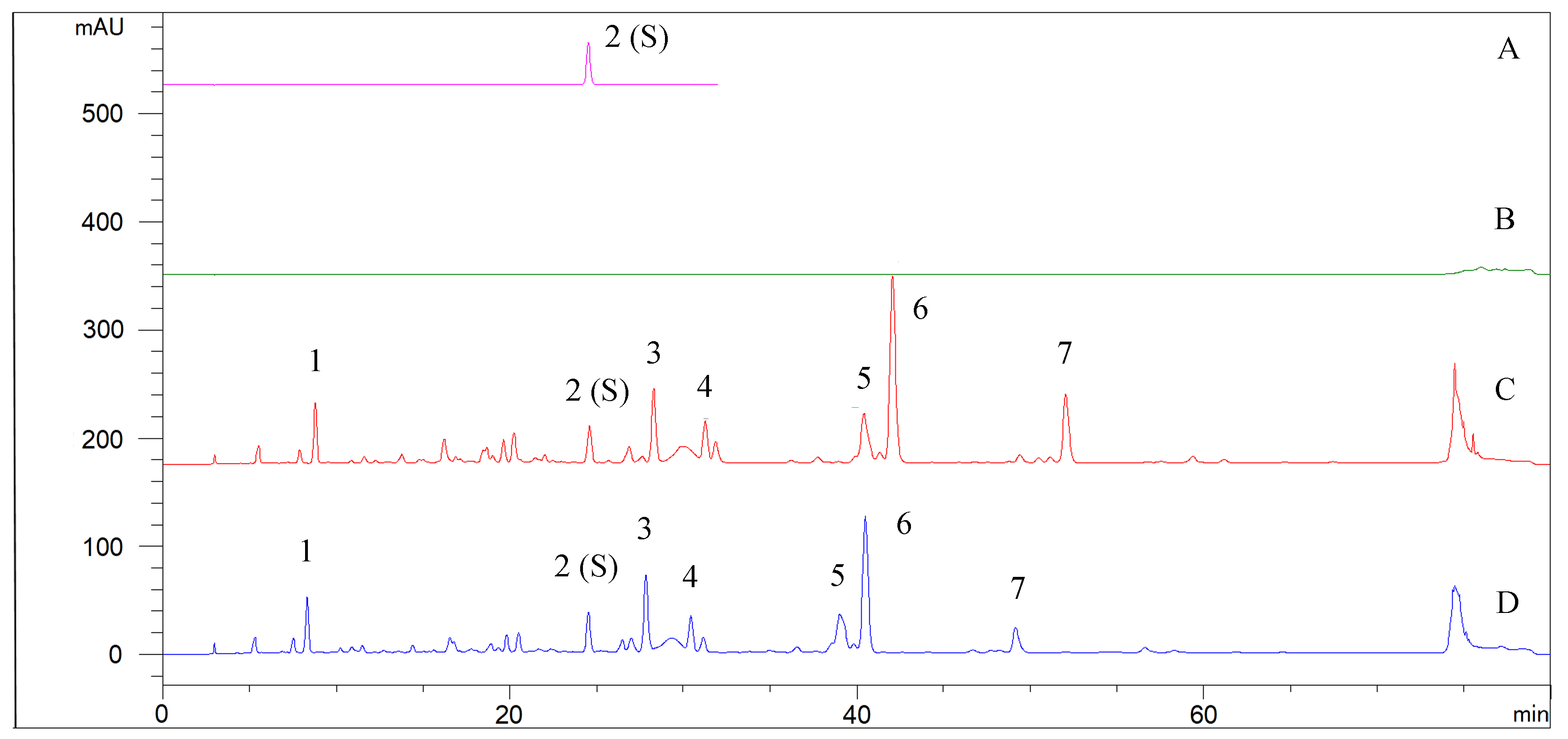

Supplement: S5 Fig — A: Standard solution of vitexin, B: solvent, C: Solution of MF reference herb, D: Sample solution of MF, 2 (S): Vitexin. (TIF) [file pone.0235533.s005.tif]
