# Supplementary material for: Integration of molecular networking and fingerprint analysis for studying constituents in Microctis Folium
Source: PLoS One. 2020 Jul 7;15(7):e0235533. doi: 10.1371/journal.pone.0235533 (PMC7340309; doi:10.1371/journal.pone.0235533)
Supplement: S1 Table — (DOCX) [file pone.0235533.s006.docx]

S1 Table Sample information

| Batch No. | Date | Season | Location | Latitude | Longitude | Altitude |
| --- | --- | --- | --- | --- | --- | --- |
|  |  |  |  | N | E | m |
| S-01 | 201507 | Summer | Suixi, Guangdong | 110.25 | 21.38 | 14 |
| S-02 | 201606 | Summer | Suixi, Guangdong | 110.25 | 21.38 | 14 |
| S-03 | 201607 | Summer | Suixi, Guangdong | 110.25 | 21.38 | 14 |
| S-04 | 201607 | Summer | Yangchun, Guangdong | 111.78 | 22.18 | 39 |
| S-05 | 201610 | Autumn | Yangxi, Guangdong | 111.62 | 21.75 | 33 |
| S-06 | 201610 | Autumn | Yangxi, Guangdong | 111.62 | 21.75 | 33 |
| S-07 | 201610 | Autumn | Yangxi, Guangdong | 111.62 | 21.75 | 33 |
| S-08 | 201611 | Autumn | Yangchun, Guangdong | 111.78 | 22.18 | 39 |
| S-09 | 201611 | Autumn | Yangchun, Guangdong | 111.78 | 22.18 | 39 |
| S-10 | 201611 | Autumn | Yangchun, Guangdong | 111.78 | 22.18 | 39 |
| S-11 | 201611 | Autumn | Yangchun, Guangdong | 111.78 | 22.18 | 39 |
| S-12 | 201612 | Winter | Suixi, Guangdong | 110.25 | 21.38 | 14 |
| S-13 | 201612 | Winter | Suixi, Guangdong | 110.25 | 21.38 | 14 |
| S-14 | 201612 | Winter | Suixi, Guangdong | 110.25 | 21.38 | 14 |
| S-15 | 201612 | Winter | Yangxi, Guangdong | 111.62 | 21.75 | 33 |
