# Supplementary material for: Integration of molecular networking and fingerprint analysis for studying constituents in Microctis Folium
Source: PLoS One. 2020 Jul 7;15(7):e0235533. doi: 10.1371/journal.pone.0235533 (PMC7340309; doi:10.1371/journal.pone.0235533)
Supplement: S2 Table — (DOCX) [file pone.0235533.s007.docx]

S2 Table. Identified compounds’ information

| No. | RT  (min) | Chemical  Formula | Molecular  Weight | Compound Name |
| --- | --- | --- | --- | --- |
| ***Alkaloids*** | | | | |
| M024 | 2.27 | C_33_H_40_N_2_O_9_ | 608.27 | Reserpine |
| M048 | 4.08 | C_15_H_24_N_2_O_17_P_2_ | 566.06 | Uridine 5'-diphosphogalactose |
| M056 | 50.28 | C_32_H_30_N_2_O_4_ | 506.22 | Asperphenamate |
| M088 | 62.13 | C_27_H_28_N_2_O_4_ | 444.20 | Benzenepropanamide |
| M110 | 15.36 | C_17_H_20_N_4_O_6_ | 376.14 | Riboflavin |
| M113 | 18.82 | C_21_H_25_NO_4_ | 355.18 | Glaucine |
| M114 | 16.10 | C_21_H_25_NO_4_ | 355.18 | Tetrahydropalmatine |
| M120 | 15.51 | C_20_H_21_NO_4_ | 339.15 | Canadine |
| M122 | 8.91 | C_19_H_23_NO_4_ | 329.16 | Reticuline |
| M124 | 25.84 | C_18_H_19_NO_4_ | 313.13 | Feruloyltyramine |
| M126 | 2.99 | C_10_H_17_N_3_O_6_S | 307.08 | Glutathione |
| M136 | 3.34 | C_10_H_13_N_5_O_5_ | 283.09 | Vernine |
| M138 | 2.73 | C_11_H_20_N_2_O_6_ | 276.13 | L-saccharopine |
| M142 | 3.13 | C_10_H_13_N_5_O_4_ | 267.10 | Adenosine |
| M145 | 2.71 | C_9_H_13_N_3_O_5_ | 243.09 | Cytarabine |
| M149 | 5.81 | C_9_H_17_NO_5_ | 219.11 | Pantothenate |
| M152 | 19.60 | C_11_H_13_NO_3_ | 207.09 | N-Acetyl-l-phenalanine |
| M154 | 7.94 | C_11_H_12_N_2_O_2_ | 204.09 | L-Tryptophan |
| M160 | 7.96 | C_12_H_14_N_2_O_2_ | 218.11 | Abrine |
| M163 | 3.37 | C_9_H_11_NO_3_ | 181.07 | L-Tyrosine |
| M165 | 2.40 | C_6_H_14_N_4_O_2_ | 174.11 | Arginine |
| ***Anthocyanidins*** | | | | |
| M066 | 34.41 | C_22_H_22_O_12_ | 478.11 | Petunidin 3-glucoside |
| ***Coumarins*** | | | | |
| M148 | 19.02 | C_11_H_10_O_5_ | 222.05 | Fraxidin |
| M158 | 18.38 | C_10_H_8_O_4_ | 192.04 | Scopoletin |
| M167 | 12.44 | C_9_H_6_O_4_ | 178.03 | Aesculetin |
| ***Flavans*** | | | | |
| M001 | 5.70 | C_45_H_38_O_18_ | 866.21 | Procyanidin C1 |
| M029 | 35.62 | C_27_H_32_O_15_ | 596.17 | Neoeriocitrin |
| M030 | 11.40 | C_27_H_32_O_15_ | 596.17 | Naringenin 6,8-C-diglucoside |
| M039 | 33.56 | C_27_H_32_O_14_ | 580.18 | Naringin |
| M040 | 27.20 | C_27_H_32_O_14_ | 580.18 | Narirutin |
| M043 | 4.35 | C_30_H_26_O_12_ | 578.14 | Procyanidin B2 |
| M044 | 6.93 | C_30_H_24_O_12_ | 577.13 | Procyanidin A2 |
| M045 | 19.37 | C_30_H_26_O_12_ | 578.14 | Procyanidin B1 |
| M046 | 16.05 | C_30_H_22_O_12_ | 574.14 | 2,2'-bis(3,4-dihydroxyphenyl)-3',4,5',6-tetrahydroxy Spiro [2H-1- benzofuran-3,9'-3,4-dihydro-2H-furo [2,3-h]chromene]-8'-one |
| M069 | 31.09 | C_21_H_22_O_12_ | 466.11 | (2R,3R)-2-(3,4-dihydroxyphenyl)-5,7-dihydroxy-3-[(2S,3R,4S,5S, 6R) -3,4,5-trihydroxy-6-(hydroxymethyl) oxan-2-yl]oxy-2,3-  dihydrochromen-4-one |
| M070 | 20.78 | C_21_H_22_O_12_ | 466.11 | (2S,3S)-3,5,7-trihydroxy-2-[4-hydroxy-3- [(2S,3R,4S,5S,6R)-3,4,5- trihydroxy-6-(hydroxymethyl) oxan-2-yl] oxyphenyl]-2,3- dihydrochromen-4-one |
| M078 | 8.81 | C_21_H_24_O_11_ | 452.13 | (2R,3R,4S,5S,6R)-2-[[2-(3,4-dihydroxyphenyl)-5,7-dihydroxy-3,4- dihydro-2H-chromen-3-yl]oxy]-6-(hydroxymethyl)oxane-3,4,5-triol |
| M079 | 25.95 | C_21_H_22_O_11_ | 450.12 | (2R,3R)-2-(3,4-dihydroxyphenyl)-5,7-dihydroxy-3-[(2S,3R,4R,5R,6S) -3,4,5-trihydroxy-6-methyloxan-2-yl]oxy-2,3-dihydrochromen-4-one |
| M080 | 21.38 | C_21_H_22_O_11_ | 450.12 | (2S)-2-(3,4-dihydroxyphenyl)-5-hydroxy-7-[(2S,3R,4S,5S,6R)-3,4,5- trihydroxy-6-(hydroxymethyl)oxan-2-yl]oxy-2,3-dihydrochromen-4- one |
| M092 | 17.70 | C_21_H_22_O_10_ | 434.12 | 5,7-dihydroxy-2-(4-hydroxyphenyl)-6-[3,4,5-trihydroxy-6-(hydroxymethyl)  oxan-2-yl]-2,3-dihydrochromen-4-one |
| M094 | 37.07 | C_21_H_22_O_10_ | 434.12 | Naringenin-7-O-glucoside |
| M127 | 21.28 | C_15_H_12_O_7_ | 304.06 | Leucocyanidin |
| M131 | 8.57 | C_15_H_14_O_6_ | 290.08 | Catechin |
| M132 | 27.07 | C_15_H_12_O_6_ | 288.06 | Dihydrokaempferol |
| M140 | 26.11 | C_15_H_12_O_5_ | 272.07 | Naringenin |
| ***Flavones*** | | | | |
| M020 | 20.75 | C_27_H_30_O_16_ | 610.15 | 2-[3,4-bis[[(2S,3R,4S,5S,6R)-3,4,5-trihydroxy-6-(hydroxymethyl)oxan-2-yl] oxy]phenyl]-5,7-dihydroxychromen-4-one |
| M025 | 30.84 | C_28_H_32_O_15_ | 608.17 | Neodiosmin |
| M031 | 10.33 | C_27_H_30_O_15_ | 594.17 | Hexanoside of (iso)vitexin |
| M037 | 16.06 | C_27_H_30_O_15_ | 594.16 | Vicenin-2 |
| M041 | 23.70 | C_27_H_30_O_14_ | 578.16 | 5,7-dihydroxy-2-(4-hydroxyphenyl)-6-[(2S,3R,4R,5S,6R) -3,4,5- trihydroxy -6-(hydroxymethyl)oxan-2-yl]-8- [(2S,3R,4R,5R,6S)-3,4,5-trihydroxy- 6- methyloxan-2-yl]chromen-4-one |
| M042 | 40.04 | C_27_H_30_O_14_ | 578.16 | Vitexin-2''-O-rhamnoside |
| M049 | 19.69 | C_26_H_28_O_14_ | 564.15 | 5,7-dihydroxy-2-(4-hydroxyphenyl)-8-[3,4,5-trihydroxy-6-(hydroxymethyl) oxan-2-yl]-6-(3,4,5-trihydroxyoxan-2-yl) chromen-4-one |
| M050 | 18.24 | C_26_H_28_O_14_ | 564.15 | 5,7-dihydroxy-2-(4-hydroxyphenyl)-6-[3,4,5-trihydroxy-6-(hydroxymethyl) oxan-2-yl]-8-(3,4,5-trihydroxyoxan-2-yl) chromen-4-one |
| M051 | 26.38 | C_26_H_28_O_14_ | 564.15 | 6-[(2S,3R,4S,5S,6R)-4,5-dihydroxy-6-(hydroxymethyl)-3-[(2S,3R,4S,5S) -3,4,5-trihydroxyoxan-2-yl]oxyoxan-2-yl]-5,7-dihydroxy-2-  (4-hydroxy phenyl) chromen-4-one |
| M060 | 21.20 | C_21_H_20_O_11_ | 448.10 | 2-(3,4-dihydroxyphenyl)-5,7-dihydroxy-8-[(3R,4R,5R,6R)-3,4,5-trihydroxy -6-(hydroxymethyl)oxan-2-yl]chromen-4-one |
| M061 | 44.77 | C_23_H_24_O_12_ | 492.13 | 5-hydroxy-2-[2-hydroxy-3-[(2S,3R,4S,5S,6R)-3,4,5-trihydroxy-6- (hydroxymethyl)oxan-2-yl]oxyphenyl]- 7,8-dimethoxychromen-4-one |
| M076 | 26.55 | C_21_H_18_O_12_ | 462.08 | (2S,3S,4S,5R,6S)-6-[2-(3,4-dihydroxyphenyl)-5-hydroxy-4-oxochromen-7- yl]oxy-3,4,5-trihydroxyoxane-2-carboxylic acid |
| M083 | 19.99 | C_21_H_20_O_11_ | 448.10 | Homoorientin |
| M084 | 28.04 | C_21_H_20_O_11_ | 448.10 | Luteoloside |
| M087 | 36.02 | C_21_H_18_O_11_ | 446.08 | Baicalin |
| M098 | 22.26 | C_21_H_20_O_10_ | 432.11 | Isovitexin |
| M133 | 49.15 | C_15_H_10_O_6_ | 286.05 | Luteolin |
| M135 | 69.27 | C_16_H_12_O_5_ | 284.07 | Negletein |
| M141 | 63.53 | C_15_H_10_O_5_ | 270.05 | Apigenin |
| M144 | 58.55 | C_15_H_10_O_4_ | 254.06 | Chrysin |
| ***Flavonols*** | | | | |
| M003 | 12.36 | C_33_H_40_O_21_ | 772.21 | Quercetin-3-O-hexosyl(1-2)deoxyhexosyl-7-O-hexoside |
| M004 | 24.89 | C_33_H_38_O_21_ | 770.23 | Quercetin-3-Galactoside-6''-Rhamnoside-3'''-Rhamnoside |
| M005 | 40.79 | C_34_H_42_O_20_ | 770.23 | 3-[(2S,3R,4S,5R,6R)-6-[[(2R,3R,4R,5S,6S)-3,5-dihydroxy-6-methyl-4- [(2S,3R,4R,5R,6S)-3,4,5-trihydroxy-6-methyloxan-2-yl]oxyoxan-2-yl]  oxymethyl]- 3,4,5-trihydroxyoxan-2-yl]oxy-2-(3,4-dihydroxyphenyl)  -5-hydroxy-7-methoxychromen-4-one |
| M006 | 26.51 | C_34_H_42_O_20_ | 770.23 | 3-[(2S,3R,4S,5S,6R)-4,5-dihydroxy-3-[(2R,3R,4R,5R,6S)-3,4,5-  trihydroxy-6-methyloxan-2-yl]oxy-6-[[(2R,3R,4R,5R,6S)-3,4,5-trihydroxy  -6-methyloxan-2-yl]oxymethyl]oxan-2-yl]oxy-5,7-dihydroxy-2-(4-  hydroxyphenyl)-6-methoxychromen-4-one |
| M007 | 15.64 | C_33_H_40_O_20_ | 756.21 | 5,7-dihydroxy-2-[4-[(2S,3R,4S,5S,6R)-3,4,5-trihydroxy-6-(hydroxymethyl)  oxan-2-yl]oxyphenyl]-3-[(2S,3R,4S,5S,6R)-3,4,5-trihydroxy-6-[[(2R,3R,4R,  5R,6S)-3,4,5-trihydroxy-6- methyloxan-2-yl]oxymethyl]oxan-2-yl]  oxychromen-4-one |
| M008 | 36.05 | C_36_H_36_O_18_ | 756.19 | [(2R,3R,4S,5S,6R)-4,5-dihydroxy-2-[5-hydroxy-2-(4-hydroxyphenyl)-  4-oxo-7-[(2S,3R,4S,5S,6R)-3,4,5-trihydroxy-6-(hydroxymethyl)oxan-  2-yl] oxychromen-3-yl]oxy-6-(hydroxymethyl)oxan-3-yl] (E)-3-(4-  hydroxyphenyl)prop-2-enoate |
| M009 | 25.04 | C_33_H_40_O_19_ | 740.22 | 3-[4,5-dihydroxy-3-(3,4,5-trihydroxy-6-methyloxan-2-yl)oxy-6-[(3,4,5-  trihydroxy-6-methyloxan-2-yl) oxymethyl]oxan-2-yl]oxy-5,7-dihydroxy-2-  (4-hydroxyphenyl)chromen-4-one |
| M010 | 58.30 | C_36_H_36_O_17_ | 740.20 | [(2R,3S,4S,5R,6S)-6-[(2S,3R,4R,5R,6S)-2-[5,7-dihydroxy-2-(4-  hydroxyphenyl)-4-oxochromen-3-yl]oxy-4,5-dihydroxy-6-methyloxan-3-yl]  oxy-3,4,5-trihydroxyoxan-2-yl]methyl (E)-3-(4-hydroxyphenyl) prop-2-  enoate |
| M013 | 16.51 | C_34_H_42_O_20_ | 770.23 | 3-[(2S,3R,4S,5R,6R)-6-[[(2R,3R,4R,5S,6S)-3,5- dihydroxy-6- methyl-4-  [(2S,3R,4R,5R,6S)-3,4,5-trihydroxy-6-methyloxan-2-yl]oxyoxan-2-yl]  oxymethyl]-3,4,5-trihydroxyoxan-2-yl]oxy-2-(3,4-dihydroxyphenyl) -5-  hydroxy-7-methoxychromen-4-one |
| M014 | 40.10 | C_28_H_32_O_16_ | 624.17 | Narcissin |
| M015 | 34.94 | C_28_H_32_O_17_ | 640.16 | Isorhamnetin 3,4'-diglucoside |
| M016 | 11.77 | C_27_H_30_O_17_ | 626.15 | 5,7-dihydroxy-2-[3-hydroxy-4-[(2S,3R,4S,5S,6R)- 3,4,5-trihydroxy-6-  (hydroxymethyl)oxan-2-yl]oxyphenyl] -3-[(2S,3R,4S, 5S,6R)-3,4,5-  trihydroxy-6-(hydroxymethyl) oxan-2-yl]oxychromen-4-one |
| M018 | 24.86 | C_28_H_32_O_16_ | 624.17 | 5,7-dihydroxy-2-(4-hydroxy-3-methoxyphenyl)-3-[(2S,3R,4S,5R,6R)-  3,4,5-trihydroxy-6-[[(2R,3R,4R,5R,6S)- 3,4,5-trihydroxy-6-methyloxan  -2-yl]oxymethyl]oxan-2-yl] oxychromen-4-one |
| M019 | 26.13 | C_27_H_30_O_15_ | 594.16 | 3-[(2S,3R,4S,5R,6R)-3,5-dihydroxy-6-(hydroxymethyl)-4-[(2S,3R,4R,5R,  6S)-3,4,5-trihydroxy-6-methyloxan-2-yl] oxyoxan-2-yl]oxy-5,7-  dihydroxy-2-(4-hydroxyphenyl) chromen-4-one |
| M021 | 28.39 | C_27_H_30_O_16_ | 610.15 | 2-(3,4-dihydroxyphenyl)-5,7-dihydroxy-3-[(2S,3R,4S,5R,6R)- 3,4,5-  trihydroxy-6-[[(2R,3R,4R,5R,6S)- 3,4,5-trihydroxy-6-methyloxan-2-yl]  oxymethyl]oxan-2-yl] oxychromen-4-one |
| M022 | 32.83 | C_27_H_30_O_16_ | 610.15 | kaempferol-3-O-hexoxyl(1-6)hexoside |
| M023 | 23.38 | C_27_H_30_O_16_ | 610.15 | Rutin |
| M032 | 24.81 | C_27_H_30_O_15_ | 594.16 | Nicotiflorin |
| M034 | 43.34 | C_27_H_30_O_15_ | 594.16 | Datiscin |
| M036 | 45.35 | C_27_H_30_O_15_ | 594.16 | kaempferol-7-O-hexosyl(1-2)deoxyhexoside |
| M038 | 48.49 | C_30_H_26_O_13_ | 594.14 | Tiliroside |
| M065 | 38.25 | C_22_H_22_O_12_ | 478.11 | isorhamnetin-3-O-glucoside |
| M071 | 30.29 | C_21_H_20_O_12_ | 464.10 | Hyperoside |
| M072 | 28.08 | C_21_H_20_O_12_ | 464.10 | Isoquercetin |
| M073 | 28.09 | C_21_H_20_O_12_ | 464.10 | Myricitrine |
| M075 | 36.54 | C_22_H_22_O_11_ | 462.12 | 5,8-dihydroxy-2-(4-hydroxyphenyl)-7-methoxy-3-[(2S,3R,4R,5R,6S)-  3,4,5-trihydroxy-6-methyloxan-2-yl]oxychromen-4-one |
| M081 | 37.77 | C_21_H_20_O_11_ | 448.10 | Quercitrin |
| M082 | 36.11 | C_21_H_20_O_11_ | 448.10 | Astragaline |
| M085 | 32.55 | C_21_H_20_O_11_ | 448.10 | kaempferol-7-O-hexoside |
| M093 | 32.92 | C_20_H_18_O_11_ | 434.08 | Reynoutrin |
| M095 | 32.58 | C_20_H_18_O_11_ | 434.08 | Guajavarin |
| M097 | 47.21 | C_21_H_20_O_10_ | 432.11 | Afzelin |
| M102 | 36.16 | C_20_H_18_O_10_ | 418.09 | Kaempferol-3-O-alpha-L-arabinopyranoside |
| M123 | 51.87 | C_16_H_12_O_7_ | 316.06 | Isorhamnetin |
| M128 | 49.28 | C_15_H_10_O_7_ | 302.04 | Quercetin |
| M134 | 61.22 | C_15_H_10_O_6_ | 286.05 | Kaempferol |
| M168 | 38.57 | C_21_H_20_O_11_ | 448.10 | 5,7-dihydroxy-2-[4-hydroxy-3-[(2S,3R,4S,5R)- 3,4,5-trihydroxyoxan-2-  yl]oxyphenyl]-3- methoxychromen-4-one |
| ***Isoflavones*** | | | | |
| M103 | 27.83 | C_21_H_20_O_9_ | 416.11 | Puerarin |
| M033 | 15.27 | C_27_H_30_O_15_ | 594.16 | 5,7-dihydroxy-3-(4-hydroxyphenyl)-6,8-bis[3,4,5-trihydroxy-6-  (hydroxymethyl)oxan-2- yl]chromen-4-one |
| ***Lignans*** | | | | |
| M164 | 14.28 | C_9_H_8_O_4_ | 180.04 | Caffeic acid |
| M125 | 50.52 | C_15_H_18_O_8_ | 326.10 | beta-D-Glucopyranose |
| M115 | 10.32 | C_16_H_18_O_9_ | 354.10 | Chlorogenic acid |
| M054 | 26.20 | C_25_H_24_O_12_ | 516.13 | (3R,5R)-3,5-bis[[(E)-3-(3,4-dihydroxyphenyl)  prop-2-enoyl]oxy]-1,4-dihydroxycyclohexane-1-carboxylic acid |
| M017 | 24.26 | C_29_H_36_O_15_ | 624.21 | verbascoside |
| ***Lignins*** | | | | |
| M026 | 10.90 | C_28_H_38_O_13_ | 582.23 | 2-[[7-hydroxy-1-(4-hydroxy-3,5-dimethoxyphenyl)- 3-(hydroxymethyl)-6,8-  dimethoxy-1,2,3,4- tetrahydronaphthalen-2-yl]methoxy]-6-(hydroxymethyl)  oxane-3,4,5-triol |
| M027 | 24.06 | C_28_H_36_O_13_ | 580.22 | (2S,3R,4S,5S,6R)-2-[4-[(3S,3aR,6S,6aR)-3-(4- hydroxy-3,5-  dimethoxyphenyl)-1,3,3a,4,6,6a-hexahydrofuro[3,4-c]furan-6-yl]  -2,6-dimethoxyphenoxy]-6-(hydroxymethyl)oxane-3,4,5-triol |
| M028 | 50.47 | C_30_H_28_O_13_ | 596.15 | ((2R,3S,4S,5R,6S)-6-(4-((E)-3-(3,4-dihydroxyphenyl)acryloyl)-2,3-  dihydroxyphenoxy)-3,4,5- trihydroxytetrahydro-2H-pyran-2-yl) methyl  (E)-3-(4-hydroxyphenyl)acrylate |
| M035 | 45.87 | C_23_H_32_O_15_ | 548.17 | ((2R,3S,4S,5R,6R)-6-(((2R,3S,4S,5R)-3,4-dihydroxy-2,5-bis  (hydroxymethyl)tetrahydrofuran-2-yl)oxy)-3,4,5- trihydroxytetrahydro  -2H-pyran-2-yl)methyl(E)-3-(4-hydroxy-3,5-dimethoxyphenyl)acrylate |
| M052 | 16.52 | C_26_H_36_O_12_ | 540.22 | 2-[3-[4-[1,3-dihydroxy-1-(4-hydroxy-3-methoxyphenyl) propan-2-yl]  oxy-3-methoxyphenyl]propoxy]-6-(hydroxymethyl)oxane-3,4,5-triol |
| M053 | 25.23 | C_26_H_34_O_11_ | 522.21 | 2-(hydroxymethyl)-6-[5-[3-(hydroxymethyl)-5-(3-hydroxypropyl)-7-  methoxy-2,3-dihydro-1- benzofuran-2-yl]-2-methoxyphenoxy] oxane-  3,4,5-triol |
| M101 | 24.72 | C_22_H_26_O_8_ | 418.16 | 4-[(3R,3aR,6S,6aR)-6-(4-hydroxy-3,5-dimethoxyphenyl)- 1,3,3a,4,6,6a-  hexahydrofuro[3,4-c]furan-3-yl]-2,6- dimethoxyphenol |
| M106 | 22.20 | C_17_H_20_O_9_ | 368.11 | (1R,3R,4S,5R)-1,3,4-Trihydroxy-5-{[(2E)-3-(4-hydroxy-3-methoxyphenyl)  -2-propenoyl]oxy}cyclohexanecarboxylic acid |
| M107 | 11.31 | C_17_H_22_O_10_ | 386.12 | 1-O-b-D-glucopyranosyl sinapate |
| M108 | 9.46 | C_17_H_24_O_9_ | 372.14 | (2R,3S,4S,5R,6S)-2-(hydroxymethyl)-6-[4-[(E)-3-hydroxyprop-1-enyl]  -2,6-dimethoxyphenoxy]oxane-3,4,5-triol |
| M118 | 29.85 | C_19_H_18_O_6_ | 342.11 | 1,3-Cyclobutanedicarboxylic acid |
| M119 | 22.32 | C_20_H_22_O_6_ | 358.14 | 5-[6-(3-hydroxy-4-methoxyphenyl)-1,3,3a,4,6,6a- hexahydrofuro[3,4-c]  furan-3-yl]-2-methoxyphenol |
| M147 | 22.04 | C_11_H_12_O_5_ | 224.07 | Sinapic acid |
| M150 | 14.39 | C_10_H_12_O_5_ | 212.07 | Vanillactic acid |
| M153 | 13.17 | C_15_H_16_O_9_ | 340.08 | Sinapoyl malate |
| M155 | 0.12 | C_10_H_10_O_4_ | 194.06 | Isoferulic acid |
| M157 | 9.19 | C_11_H_12_O_3_ | 192.08 | Myristicin |
| M166 | 8.23 | C_10_H_10_O_4_ | 194.06 | Ferulic acid |
| ***Others*** | | | | |
| M067 | 23.29 | C_20_H_28_O_10_ | 428.17 | 4-Vinylphenyl 6-O-(6-deoxy-alpha-L-mannopyranosyl) -beta-D-  glucopyranoside |
| M068 | 15.21 | C_19_H_26_O_12_ | 446.14 | Methyl 2-{[6-O-(beta-D-xylopyranosyl)-beta-D- glucopyranosyl]oxy}  benzoate |
| M074 | 22.38 | C_23_H_22_O_8_ | 426.13 | (2R,6aR)-6a-hydroxy-2-(3-hydroxyprop-1-en-2-yl)- 8,9-dimethoxy-  1,2,12,12a-tetrahydrochromeno[3,4-b] furo[2,3-h]chromen-6(6aH) -one |
| M086 | 22.82 | C_20_H_30_O_10_ | 430.18 | 2-Phenylethyl 6-O-(6-deoxy-alpha-L-mannopyranosyl)- beta-D-  glucopyranoside |
| M090 | 45.15 | C_19_H_34_O_10_ | 422.22 | (2R,3S,4S,5R,6R)-2-[[(2R,3R,4R)-3,4-dihydroxy-4-(hydroxymethyl) oxolan  -2-yl]oxymethyl]-6-oct-1-en-3-yloxyoxane-3,4,5-triol |
| M096 | 18.30 | C_19_H_28_O_10_ | 416.17 | 2-Phenylethyl 2-O-[(2S,3R,4R)-3,4-dihydroxy-4-(hydroxymethyl)  tetrahydro-2-furanyl]-beta-D- glucopyranoside |
| M100 | 12.68 | C_18_H_26_O_10_ | 402.15 | (2R,3R,4S,5S,6S)-2-(benzyloxy)-6-((((2R,3R,4R)- 3,4-dihydroxy-4-  (hydroxymethyl)tetrahydrofuran-2- yl)oxy)methyl)tetrahydro-2H-pyran-  3,4,5-triol |
| M105 | 14.09 | C_18_H_28_O_9_ | 388.17 | Cyclopentaneacetic acid |
| M121 | 15.62 | C_19_H_24_O_6_ | 348.16 | Naphtho[2,3-b]furan-9(4H)-one |
| M130 | 66.88 | C_18_H_30_O_4_ | 310.21 | 9(S)-HpOTrE |
| M137 | 48.99 | C_18_H_30_O_3_ | 294.22 | 9S-Hydroxy-10E,12Z,15Z-octadecatrienoic acid |
| M139 | 70.63 | C_18_H_28_O_3_ | 292.20 | 9-OxoOTrE |
| M146 | 13.46 | C_12_H_18_O_4_ | 226.12 | {(1R,2R)-2-[(2Z)-5-Hydroxy-2-penten-1-yl]-3- oxocyclopentyl} acetic acid |
| M159 | 3.02 | C_6_H_8_O_7_ | 192.03 | Citric acid |
| M161 | 36.52 | C_9_H_16_O_4_ | 188.10 | Azelaic acid |
| ***Phenols*** | | | | |
| M117 | 8.94 | C_15_H_22_O_9_ | 346.13 | (2R,3S,4S,5R,6S)-2-(hydroxymethyl)-6-(3,4,5- trimethoxyphenoxy)  oxane-3,4,5-triol |
| M116 | 8.96 | C_16_H_18_O_9_ | 354.10 | 5,7-dihydroxy-2-methyl-8-[(2S,3R,4R,5S,6R)-3,4,5- trihydroxy-6-(  hydroxymethyl)oxan-2-yl]chromen-4-one |
| M077 | 6.23 | C_19_H_26_O_13_ | 462.14 | beta-D-Fructofuranosyl 6-O-(4-hydroxybenzoyl)- alpha-D-  glucopyranoside |
| M064 | 10.15 | C_19_H_28_O_11_ | 432.16 | (2R,3S,4S,5R,6R)-2-[[(2R,3R,4R)-3,4-dihydroxy-4-(hydroxymethyl)oxolan  -2-yl]oxymethyl]-6-[2-(4- hydroxyphenyl)ethoxy]oxane-3,4,5-triol |
| ***Polyols*** | | | | |
| M058 | 27.83 | C_24_H_42_O_12_ | 522.27 | 6-Hydroxy-2,6,10,10-tetramethyl-1-oxaspiro[4.5]dec-8-yl6-O-[(2R,3R,4R)  -3,4-dihydroxy-4-(hydroxymethyl) tetrahydro-2-furanyl]- beta-D-  glucopyranoside |
| M063 | 10.20 | C_18_H_34_O_12_ | 442.21 | 3-Hydroxy-3-(hydroxymethyl)-4-methylpentyl 6-O-[(2S,3R,4R)-  3,4-dihydroxy-4- |
| M091 | 10.30 | C_18_H_24_O_12_ | 432.13 | 2-Methyl-4-oxo-4H-pyran-3-yl 6-O-(4-carboxy-3- hydroxy-3-  methylbutanoyl)-beta-D-glucopyranoside |
| M104 | 9.46 | C_19_H_32_O_8_ | 388.21 | 4-hydroxy-3,3,5-trimethyl-4-[(E)-3-[3,4,5-trihydroxy-6-(hydroxymethyl)  oxan-2-yl]oxybut-1-enyl] cyclohexan-1-one |
| M151 | 2.90 | C_6_H_10_O_8_ | 210.04 | Galactarate |
| M156 | 2.81 | C_11_H_20_NO_8_ | 294.12 | maltitol |
| M162 | 2.82 | C_6_H_14_O_6_ | 182.08 | sorbitol |
| ***Steroids*** | | | | |
| M143 | 20.69 | C_19_H_30_O_2_ | 290.22 | Epiandrosterone |
| M047 | 11.86 | C_29_H_44_O_8_ | 520.30 | 4-((3S,10S,13R,14S,17R)-14-hydroxy-10,13-dimethyl-3-(((2R,3R,4S, 5R,  6S)-3,4,5-trihydroxy-6- methyltetrahydro-2H-pyran-2-yl)oxy)  hexadecahydro-1H-cyclopenta[a]phenanthren-17-yl)furan-2(5H)-one |
| M002 | 68.01 | C_39_H_64_O_13_ | 740.43 | beta-D-Glucopyranoside, 3,23-dihydroxyspirostan-6-yl 6-deoxy-4-O-(6-deoxy-alpha-L-mannopyranosyl)- |
| ***Terpenoids*** | | | | |
| M011 | 42.42 | C_36_H_58_O_11_ | 666.40 | 1-O-[(2alpha,3beta,5xi,6beta,9xi,18xi)-2,3,6,23- Tetrahydroxy-28-  oxoolean-12-en-28-yl]-beta-D- glucopyranose |
| M012 | 65.16 | C_36_H_58_O_10_ | 650.40 | 1-O-[(2alpha,3beta,5xi,9xi,18xi)-2,3,23-Trihydroxy-28- oxoolean-12-  en-28-yl]-beta-D-glucopyranose |
| M055 | 63.47 | C_22_H_38_O_10_ | 462.25 | 2-(4-Methyl-3-cyclohexen-1-yl)-2-propanyl-6-O-(6-deoxy- alpha-L- mannopyranosyl)- beta-D-glucopyranoside |
| M057 | 30.65 | C_24_H_40_O_11_ | 504.26 | 2-Cyclohexen-1-one |
| M059 | 54.03 | C_21_H_36_O_10_ | 448.23 | (6,6-Dimethylbicyclo[3.1.1]hept-2-yl)methyl 6-O-[(2R,3R,4R)-3,4-  dihydroxy-4-(hydroxymethyl)tetrahydro-2-furanyl]-beta-D-  glucopyranoside |
| M062 | 6.98 | C_21_H_32_O_10_ | 444.20 | 1-O-{(2E,4E)-5-[(1R,3S,5S,8S)-3,8-Dihydroxy-1,5- dimethyl-6-oxabicyclo  [3.2.1]oct-8-yl]-3-methyl-2,4- pentadienoyl}-beta-D-glucopyranose |
| M089 | 16.22 | C_21_H_32_O_10_ | 444.20 | 2,4-Pentadienoic acid |
| M109 | 18.14 | C_19_H_32_O_8_ | 388.21 | (2R)-4-[(1S)-1-Hydroxy-2,6,6-trimethyl-4-oxo-2- cyclohexen-1-yl]-2-  butanyl beta-D-glucopyranoside |
| M111 | 29.29 | C_19_H_34_O_7_ | 374.23 | 4-(4-Hydroxy-2,6,6-trimethyl-1-cyclohexen-1-yl)-2- butanyl beta-D-  glucopyranoside |
| M112 | 29.59 | C_19_H_32_O_7_ | 372.21 | 4-(2,6,6-Trimethyl-4-oxo-2-cyclohexen-1-yl)-2-butanylbeta-D- glucopyranoside |
| M129 | 41.70 | C_20_H_40_O | 296.31 | Phytol |
| ***Xanthones*** | | | | |
| M099 | 16.75 | C_19_H_18_O_11_ | 422.08 | Mangiferin |
