# Supplementary material for: Integration of molecular networking and fingerprint analysis for studying constituents in Microctis Folium
Source: PLoS One. 2020 Jul 7;15(7):e0235533. doi: 10.1371/journal.pone.0235533 (PMC7340309; doi:10.1371/journal.pone.0235533)
Supplement: S3 Table — (DOCX) [file pone.0235533.s008.docx]

S3 Table. Identification of compounds in MF.

| No. | [M+H]^+^/ppm  (Error) | [M-H]^－^/ppm  (Error) | Main fragment ions(*m/z*) | |
| --- | --- | --- | --- | --- |
|  |  |  | Positive ion mode | Negative ion mode |
| M001 |  | 865.20 (4) |  | 577.16[M－C_15_H_13_O_6_]^－^, 407.08[M－H－C_15_H_13_O_6_－C_8_H_8_O_3_－H_2_O]^－^, 289.08[M－C_15_H_13_O_6_－C_15_H_12_O_6_]^－^, 287.06[M－2C_15_H_13_O_6_]^－^, 125.03[ M－H－2C_15_H_13_O_6_－C_7_H_4_O_3_－C_2_H_2_]^－^ |
| M002 |  | 739.43 (6) |  | 785.43[M+COOH]^－^, 593.37[M－Rha]^－^,145.05[Fucose]^－^ |
| M003 |  | 771.20 (5) |  | 609.15[M－Glu]^－^, 462.08[M－Rha－Glu]^－^, 301.04[M－H－Rha－Glu－Glu]^－^ |
| M004 | 771.24(1) |  | 479.12[M+H－Rha－Rha]^+^, 317.07 [M+H－Rha－Rha－Gal]^+^ |  |
| M005 | 771.23(0) | 769.22 (5) | 479.12[M+H－Rha－Rha]^+^, 317.07 [M+H－Rha－Rha－Gal]^+^ | 314.04[M－H－Rha－Rha－Gal]^－^, 299.02[M－H－Rha－Rha－Gal－CH_3_]^－^, 271.02[M－H－Rha－Rha－Gal－CH_3_－CO]^－^, 165.02[M－H－Rha－Rha－Gal－C_8_H_5_O_3_]^－^ |
| M006 |  | 769.22 (10) |  | 314.04[M－H－Rha－Rha－Glu]^－^, 299.02[M－H－Rha－Rha－Glu－CH_3_]^－^, 271.02[M－H－Rha－Rha－Glu－CH_3_－CO]^－^,243.03[M－H－Rha－Rha－Glu－CH_3_－2CO]^－^ |
| M007 | 757.22(1) | 755.20 (2) | 449.11[M+H－Glu－Rha] ^+^, 287.05[M+H－2Glu－Rha] ^+^, | 593.15[M－H－Rha]^－^, 447.09[M－H－Glu－Rha]^－^, 285.04[M－H－2Glu－Rha]^－^ |
| M008 | 757.20(6) |  | 449.11[M+H－Glu－C_9_H_7_O_2_]^+^,309.10[Glu+C_9_H_7_O_2_]^+^, 287.06[M+H－2Glu－C_9_H_7_O_2_] ^+^, 147.04[C_9_H_7_O_2_]^+^ |  |
| M009 | 741.22(0) | 739.21 (4) | 449.11[M+H－2Rha] ^+^,287.06[M+H－Glu－2Rha] ^+^ | 284.03[M－H－Glu－2Rha]^－^,255.03[M－H－Glu－2Rha－CHO]^－^,227.03[M－H－Glu－2Rha－CHO－CO]^－^ |
| M010 | 741.20 (6) | 739.19 (4) | 309.10[Glu+C_9_H_7_O_2_]^+^, 287.06[M+H－C_9_H_7_O_2_－Glu－Rha] ^+^, 147.04[C_9_H_7_O_2_]^+^ | 593.15[M－C_9_H_7_O_2_]^－^, 284.03[M－H－C_9_H_7_O_2_－Glu－Rha]^－^, 255.03[M－H－C_9_H_7_O_2_－Glu－Rha－CHO]^－^, 227.03[M－H－C_9_H_7_O_2_－Glu－Rha－CHO－CO]^－^ |
| M011 |  | 665.39(0) |  | 711.40[M+COOH]^－^, 503.34[M－Glu]^－^ |
| M012 |  | 649.40(4) |  | 695.40[M+COOH]^－^, 487.34[M－Glu]^－^ |
| M013 | 641.17 (6) |  | 317.07[M+H－2Glu] ^+^ |  |
| M014 | 625.18 (7) |  | 317.07[M+H－Rutinose] ^+^ |  |
| M015 |  | 639.16 (3) |  | 623.16[M－OH]^－^, 315.05[M－2Glu]^－^, 314.04[M－H－2Glu]^－^, 300.03[M－2Glu－CH_3_]^－^, 299.02[M－H－2Glu－CH_3_]^－^, 271.03[M－H－2Glu－CH_3_－CO]^－^, 255.03[M－H－2Glu－CH_3_O－CO]^－^, 243.03[M－H－2Glu－CH_3_－2CO]^－^ |
| M016 |  | 625.14 (3) |  | 463.09[M－H－Glu]^－^, 301.04[M－2Glu]^－^, 300.03[M－H－2Glu]^－^, 179.00[Glu－H]^－^ |
| M017 |  | 623.20 (6) |  | 461.17[M－C_9_H_7_O_3_]^－^, 161.02[C_9_H_7_O_4_－H_2_O]^－^ |
| M018 | 625.18 (3) | 623.16 (7) | 317.06[M+H－Rha－Gal]^+^, 302.04[M+H－Rha－Gal－CH_3_]^+^ | 669.17 [M+COOH]^－^, 315.05[M－Rha－Gal]^－^, 314.04[M－H－Rha－Gal]^－^, 300.03[M－H－Rha－Gal－CH_3_]^－^, 299.02[M－H－Rha－Gal－CH_3_]^－^, 255.02[M－H－Rha－Gal－CH_3_O－CO]^－^ |
| M019 | 617.15 (4) |  | 617.15[M+Na]^+^, 331.10[Rha+Glu+Na－H_2_O] ^+^, 308.03[M+ Na－Rha－Glu] ^+^ |  |
| M020 | 611.16 (1) |  | 449.11[M+H－Glu] ^+^, 287.06[M+H－2Glu] ^+^ |  |
| M021 | 611.16 (8) |  | 303.05[M+ H－Rha－Glu] ^+^ |  |
| M022 |  | 609.15(2) |  | 429.08[M－Glu－H_2_O]^－^, 284.03[M－H－2Glu]^－^ |
| M023 |  | 609.15 (6) |  | 301.03[M－Rutinose]^－^, 300.02[M－H－Rutinose]^－^, |
| M024 | 609.28 (3) |  | 397.21[M+H－C_10_H_12_O_5_]^+^, 195.07[M+H－C_23_H_30_N_2_O_5_]^+^, 174.09[M+H－C_22_H_29_O_8_－CH_3_]^+^ |  |
| M025 | 609.18 (4) |  | 463.13[M+H－ Fucose]^+^, 301.07[M+H－Neohesperidose]^+^, 286.05[M+H－Neohesperidose－CH_3_]^+^ |  |
| M026 | 583.24(1) | 581.22 (3) | 267.12[M－Glu－C_8_H_8_O_3_]^+^, 249.11[M－Glu－C_8_H_8_O_3_－H_2_O]^+^, 187.08[M－Glu－C_8_H_8_O_3_－H_2_O－2CH_3_O]^+^, 159.08[M－Glu－C_8_H_8_O_3_－H_2_O－CO－2CH_3_O]^+^ | 627.23 [M+COOH]^－^, 419.17[M－Glu ]^－^ |
| M027 | 598.25 (0) | 625.21 (1) | 401.16[M－Glu－O]^+^, 265.11[M－Glu－C_8_H_8_O_3_]^+^, 205.09[M－Glu－C_8_H_8_O_3_－CHO－CH_3_O]^+^, 167.07[M+H－Glu－C_8_H_8_O_3_－C_5_H_7_O_2_]^+^, | 417.15[M－H－Glu]^－^, 402.13[M－H－Glu－CH_3_]^－^, 387.11[M－H－Glu－CH_3_]^－^, 181.05[M－H－Glu－C_9_H_9_O_4_－C_4_H_6_]^－^, 166.03[M－H－Glu－C_9_H_9_O_4_－C_4_H_6_－CH_3_]^－^ |
| M028 | 597.12 (6) |  | 309.07[M+H－C_15_H_11_O_6_]^+^, 291.06[M+H－C_15_H_11_O_6_－H_2_O]^+^, 289.04[M+H－C_15_H_17_O_7_]^+^, 147.03[M+H－C_15_H_11_O_6_－Glu]^+^ |  |
| M029 |  | 595.17 (8) |  | 459.12[M－H－C_8_H_8_O_2_]^－^, 287.06[M－H－Rha－Glu]^－^,151.00[M－H－Rha－Glu－C_8_H_8_O_2_]^－^,135.04[C_8_H_8_O_2_－H]^－^ |
| M030 |  | 595.17 (3) |  | 475.12[M－H－C_4_H_8_O_4_]^－^, 385.09[M－H－Glu－C_2_H_4_O_2_]^－^, 355.08[M－H－Glu－C_3_H_6_O_3_]^－^ |
| M031 | 595.17 (5) |  | 433.11[M+H－Glu]^+^, 415.10[M+H－Glu－H_2_O]^+^, 397.09[M+H－Glu－2H_2_O]^+^, 337.07[M+H－Glu－2H_2_O－C_2_H_4_O_2_]^+^, 313.07[M+H－Glu－C_4_H_8_O_4_]^+^ |  |
| M032 | 595.17 (3) |  | 449.11[M+H－Rha]^+^, 287.06[M+H－Rutinose]^+^ |  |
| M033 | 595.16 (1) |  | 457.11[M+H－C_4_H_8_O_4_－H_2_O]^+^, 379.08[M－Glu－CHO－2H_2_O]^+^, 337.07[M+H－Glu－C_3_H_6_O_3_－H_2_O]^+^, 325.07[M+H－Glu－C_4_H_8_O_4_]^+^, 295.06[M+H－2Glu]^+^ |  |
| M034 |  | 593. 15 (5) |  | 285.04[M－Rutinose]^－^, 284.00[M－H－Rutinose]^－^, 151.00[M－H－Rutinose－C_8_H_5_O_2_]^－^, 125.00[M－Rutinose－C_6_H_4_O_2_－CO_2_－H_2_O]^－^ |
| M035 |  | 547.17 (6) |  | 593.17[M+COOH]^－^, 265.07[M－H－Fru－C_4_H_7_O_4_]^－^, 223.06[M－Fru－Glu]^－^, 205.05[M－Fru－Glu－H_2_O]^－^, 190.03[M－Fru－Glu－H_2_O－CH_3_]^－^, 164.05[M－Fru－Glu－CO_2_－CH_3_]^－^ |
| M036 |  | 593.15 (7) |  | 285.04[M－Rha－Glu]^－^ |
| M037 |  | 593.13 (2) |  | 503.11[M－H－C_3_H_6_O_3_]^－^, 473.10[M－H－C_4_H_8_O_4_]^－^, 383.07[M－H－Glu－C_2_H_4_O_2_]^－^, 353.06[M－H－Glu－C_3_H_6_O_3_]^－^ |
| M038 | 595.15 (3) | 593.13 (8) | 309.10[M+H－C_15_H_10_O_6_支链]^+^,287.05[M+H－C_15_H_7_O_7_]^+^, 147.04[M+H－C_15_H_10_O_6_－Glu]^+^, 119.05[M+H－C_15_H_10_O_6_－Glu－CO]^+^ | 285.04[M－Glu－C_9_H_7_O_2_]^－^, 284.03[M－H－Glu－C_9_H_7_O_2_]^－^, 255.03[M－H－Glu－C_9_H_7_O_2_－CHO]^－^ |
| M039 |  | 579.17 (10) |  | 459.11[M－H－C_8_H_8_O]^－^, 271.06[M－Neohesperidose]^－^, 151.00[M－Neohesperidose－C_8_H_8_O]^－^ |
| M040 |  | 579.17 (5) |  | 579.17[M－H]^－^, 459.12[M－H－C_8_H_8_O]^－^, 271.06[M－Rutinose]^－^, 193.02[M－Rha－C_4_H_7_O－C_8_H_8_O]^－^, 151.00[M－Rutinose－C_8_H_8_O]^－^ |
| M041 | 579.17 (1) | 577.16 (5) | 457.11[M+H－H_2_O－C_4_H_8_O_3_]^+^, 379.08[M－Glu－2H_2_O]^+^, 337.07[M+H－Glu－H_2_O－C_3_H_6_O_2_]^+^, 325.07[M+H－Glu－C_4_H_8_O_3_]^+^, 121.03[C_9_H_6_O_3_+ H－C_2_H_2_O]^+^ | 457.12[M－C_4_H_9_O_4_]^－^,383.08[M－Glu－CHOH－CH_3_]^－^, 353.07[M－Glu－2CHOH－CH_3_]^－^ |
| M042 | 579.17 (4) |  | 433.11[M+H－Rha]^+^, 415.10[M+H－Rha－H_2_O]^+^, 397.09[M+H－Rha－2H_2_O]^+^,313.07[M+H－Rha－C_4_H_8_O_4_]^+^,283.06[M+H－Rha－C_5_H_10_O_5_]^+^ |  |
| M043 | 579.15(5) | 577.14 (8) | 601.13[M+Na]^+^, 449.08[M+Na－C_8_H_8_O_3_]^+^, 431.07[M+Na－C_8_H_8_O_3_－H_2_O]^+^, 311.05[M+Na－C_15_H_14_O_6_]^+^ | 409.09[M－H－C_8_H_8_O_4_]^－^, 287.05[M－H－C_15_H_14_O_6_]^－^, 163.04[M－H－C_15_H_13_O_6_－C_6_H_4_O_2_－H_2_O]^－^, 139.04[M－C_15_H_13_O_6_－C_6_H_4_O_2_－C_2_H_2_O]^－^, 127.04[M－H－C_15_H_13_O_6_－C_6_H_4_O_2_－CHO]^－^, 123.04[M－H－C_15_H_13_O_6_－C_7_H_5_O_3_－CO]^－^ |
| M044 | 577.13 (10) |  | 425.09[M+H－C_8_H_8_O_3_]^+^,287.05,123.04[M+H－C_22_H_16_O_9_－CHOH]^+^ |  |
| M045 |  | 577.13 (4) |  | 425.09[M－H－C_8_H_8_O_3_]^－^, 407.08[M－H－C_8_H_8_O_3_－H_2_O]^－^, 289.07[M－C_15_H_13_O_6_]^－^, 125.03[ M－C_15_H_13_O_6_－C_9_H_8_O_3_]^－^ |
| M046 |  | 573.10 (1) |  | 477.07[M－H－C_5_H_5_O_2_]^－^, 285.04[M－H－C_13_H_15_O_5_－CHOH]^－^, 283.03[M－H－C_15_H_12_O_5_－H_2_O]^－^ |
| M047 |  | 519.30 (9) |  | 565.30[M+COOH]^－^, 387.25[M－H－C_4_H_8_O_3_－CO]^－^ |
| M048 |  | 565.05 (4) |  | 384.98[M－H－Glu－H_2_O]^－^, 323.03[M－H－Glu－HPO_3_]^－^, 272.96[M－H－Glu－C_4_H_3_N_2_O_2_－H_2_O]^－^, 211.00[M－H－Glu－HPO_3_－C_4_H_3_N_2_O_2_]^－^, 158.92[M－H－Glu－C_4_H_3_N_2_O_2_－C_5_H_8_O_4_]^－^ |
| M049 | 565.15 (8) | 563.14 (0) | 529.13[M+H－2H_2_O]^+^, 427.10[M+H－Ara－H_2_O]^+^, 409.09[M+H－Ara－2H_2_O]^+^, 379.08[M+H－Ara－2H_2_O－CHOH]^+^, 325.07[M+H－Ara－C_4_H_8_O_4_]^+^, 295.06[M+H－Ara－Glu]^+^, 121.03[C_4_H_8_O_4_+H]^+^ | 545.13[M－H－H_2_O]^－^, 503.12[M－H－C_2_H_4_O_2_]^－^, 473.11[M－H－C_3_H_6_O_3_]^－^, 443.10[M－C_3_H_6_O_3_－CH_2_OH]^－^, 383.08[M－C_3_H_6_O_3_－CH_2_OH－C_2_H_4_O_2_]^－^, 353.06[M－C_3_H_6_O_3_－CH_2_OH－C_3_H_6_O_3_]^－^ |
| M050 | 565.15 (5) |  | 547.15[M+H－H_2_O]^+^, 529.14[M+H－2H_2_O]^+^, 511.13[M+H－3H_2_O]^+^, 427.10[M+H－C_4_H_8_O_4_－H_2_O]^+^, 409.09[M+H－C_4_H_8_O_4_－2H_2_O]^+^, 391.08[M+H－C_4_H_8_O_4_－3H_2_O]^+^, 379.09[M+H－C_5_H_10_O_5_－2H_2_O]^+^, 337.07[M+H－Ara－2H_2_O－C_3_H_6_O_2_]^+^, 325.07[M+H－Ara－C_4_H_8_O_4_]^+^, 295.06[M+H－Ara－Glu]^+^, 195.03[M+H－Ara－Glu－C_3_H_2_O－H_2_O－CO]^+^, 121.03[C_4_H_8_O_4_+H]^+^ |  |
| M051 | 565.16 (6) |  | 433.11[M+H－Ara]^+^, 337.07[M+H－Ara－2H_2_O－CH_2_OH－CHO]^+^, 313.07[M+H－Ara－C_4_H_8_O_4_]^+^, 283.06[M+H－Ara－Glu]^+^, |  |
| M052 | 539.21 (1) | 539.21(6) | 331.16[M+H－Glu－H_2_O－CO]^+^, 207.10[M+H－Glu－H_2_O－C_8_H_9_O_3_]^+^, 137.06[M+H－Glu－C_2_H_4_O－C_10_H_13_O_4_]^+^ | 585.22[M+COOH]^－^, 491.19[M－CH_3_O－H_2_O ]^－^, 343.14[M－Glu－2OH]^－^, 195.07[M－Glu－C_10_H_12_O_2_－H_2_O]^－^, 165.06[M－Glu－C_10_H_12_O_2_－CH_2_OH－OH]^－^, 150.04[M－Glu－C_10_H_12_O_2_－CH_2_OH－OH－CH_3_]^－^ |
| M053 | 540.24 (3) |  | 331.15[M+H－Glu－CO]^+^,313.14[M+H－Glu－H_2_O－CO]^+^, 287.13[M+H－Glu－H_2_O－CO－C_2_H_2_]^+^, 151.07[M－Glu－CO－C_10_H_12_O_3_]^+^, 137.06[M+H－Glu－C_10_H_12_O_3_－CH_3_－CO]^+^ |  |
| M054 |  | 515.12 (5) |  | 353.09[M－C_9_H_7_O_3_]^－^, 191.05[M－H－C_9_H_7_O_3_－C_6_H_9_O_5_]^－^, 179.03[M－C_9_H_7_O_3_－C_7_H_10_O_5_]^－^, 135.04[M－C_9_H_7_O_3_－C_7_H_10_O_5_－CO_2_]^－^ |
| M055 |  | 461.24 (3) |  | 507.25[M+COOH]^－^, 315.18[M－Rha]^－^, 161.05[M－Rha－C_10_H_17_－OH]^－^ |
| M056 | 507.23 (159) |  | 256.13[M+H－C_16_H_14_NO_2_]^+^, 238.12[M－C_16_H_14_NO_3_]^+^, 224.11[M－C_17_H_16_NO_3_]^+^, 117.07[M+H－C_16_H_14_NO_3_－C_7_H_5_O－NH_3_]^+^, 105.03[M－C_16_H_14_NO_3_－C_9_H_11_N]^+^ |  |
| M057 | 505.26 (1) | 503.25 (6) | 211.17[M+H－Xyl－Glu]^+^,193.16[M+H－Xyl－Glu－H_2_O]^+^, 135.12[M－Xyl－Glu－C_2_H_4_O－CO－2CH_3_]^+^, 109.10[M－Xyl－Glu－C_4_H_8_O－CO]^+^ | 549.25[M+COOH]^－^, 371.21[M－Xyl]^－^, 161.04[M－H－Xyl－C_13_H_21_O_2_]^－^ |
| M058 | 523.28(3) | 521.26 (4) | 505.27[M+H－H_2_O]^+^, 211.17[M－Apiose－Glu]^+^, 193.16[M－Apiose－Glu－H_2_O]^+^, 155.11[M+H－Apiose－Glu－C_2_H_3_O－2CH_3_]^+^, | 567.27[M+COOH]^－^, 389.22[M－Apiose]^－^, |
| M059 |  | 447.22(0) |  | 493.23[M+COOH]^－^, 315.18[M－Apiose]^－^, 161.05[M－H－Apiose－C_10_H_17_O]^－^ |
| M060 |  | 447.09(6) |  | 493.10[M+COOH]^－^, 357.07[M－H－C_3_H_6_O_3_]^－^, 327.05[M－H－C_4_H_8_O_4_]^－^, 297.04[M－H－Gal]^－^ |
| M061 |  | 491.12 (4) |  | 329.07[M－Glu]^－^, 314.05[M－Glu－CH_3_]^－^, 299.02[M－Glu－2CH_3_]^－^, 201.02[M－Glu－C_6_H_8_O_3_]^－^苯环开裂 |
| M062 |  | 443.20 (2) |  | 323.15[M－H－C_4_H_8_O_4_]^－^, 305.14[M－H－C_4_H_8_O_4_－H_2_O]^－^, 281.14[M－Glu]^－^, 237.15[M－Glu－CO_2_]^－^ |
| M063 |  | 441.20 (2) |  | 487.20[M+COOH]^－^, 441.20[M－H]^－^, 309.16[M－Apiose]^－^, 251.08[M－Apiose－2CH_3_－CO]^－^, 191.06[M－Apiose－2CH_3_－CO－C_2_H_4_O_2_]^－^, 149.04[M－C_7_H_15_O_2_－Glu]^－^ |
| M064 |  | 431.16 (2) |  | 477.16 [M+COOH]^－^, 299.12[M－Apiose]^－^, 149.05[M－C_8_H_9_O－Glu]^－^, 119.05[M－C_8_H_9_O－Glu－CHOH]^－^ |
| M065 | 479.12 (4) | 477.10 (3) | 317.07[M+H－Glu]^+^, 302.04[M+H－Glu－CH_3_]^+^, 153.02[M+H－Glu－C_9_H_7_O_3_]^+^ | 314.04[M－H－Glu]^－^, 299.02[M－H－Glu－CH_3_]^－^, 285.04[M－H－Glu－CHO]^－^, 271.03[M－H－Glu－CH_3_－CO]^－^, 257.05[M－H－Glu－CHO－CO]^－^, 243.03[M－H－Glu－CH_3_－2CO]^－^ |
| M066 |  | 477.10 (7) |  | 314.04[M－H－Glu]^－^, 299.02[M－H－Glu－CH_3_]^－^, 271.03[M－H－Glu－CH_3_－CO]^－^, 243.03[M－H－Glu－CH_3_－2CO]^－^, 125.02[M－H－Glu－CH_3_－C_10_H_5_O_4_]^－^ |
| M067 |  | 427.17(8) |  | 473.17[M+COOH]^－^,307.11[M－C_4_H_8_O_3_－OH]^－^, 247.09[M－C_4_H_8_O_3_－OH－C_2_H_4_O_2_]^－^, 163.06[M－C_8_H_7_－Glu ]^－^, 119.05[M－Rutinose]^－^ |
| M068 | 447.14 (0) |  | 469.13[M+Na]^+^, 317.09[M+H－C_2_H_2_－C_4_H_8_O_3_]^+^ |  |
| M069 | 467.12 (7) |  | 305.07[M+H－Glu]^+^, 287.05[M+H－Glu－H_2_O]^+^, 259.06[M+H－Glu－H_2_O－CO]^+^, 231.07[M+H－Glu－H_2_O－2CO]^+^, 153.02[M+H－Glu－C_8_H_7_O_3_]^+^RDA, 123.04[M+H－Glu－C_7_H_4_O_4_－CO]^+^ |  |
| M070 |  | 465.10 (4) |  | 437.11[M－H－CO]^－^, 303.05[M－H－Glu]^－^, 285.04[M－H－Glu－H_2_O]^－^, 275.06[M－H－Glu－CO]^－^,152.01[M－Glu－C_8_H_7_O_3_]^－^ |
| M071 |  | 463.09 (6) |  | 300.03[M－H－Glu]^－^,271.03[M－H－Glu－CHO]^－^,255.03[M－Glu－CO－H_2_O]^－^,243.03[M－H－Glu－CHO－CO]^－^,151.00[M－H－Glu－C_8_H_5_O_3_]^－^ RDA |
| M072 | 465.10 (2) |  | 303.05[M+H－Glu]^+^, 153.02[M+H－Glu－C_8_H_5_O_3_]^+^RDA |  |
| M073 |  | 463.09 (2) |  | 316.02[M－H－Rha]^－^, 271.03[M－Rha－CO－H_2_O]^－^, 259.03[M－H－Rha－CO－CHO]^－^, 214.03[M－H－Rha－3CO－H_2_O ]^－^, 179.00[M－H－Rha－C_7_H_5_O_3_ ]^－^, 151.00[M－H－Rha－C_8_H_5_O_4_ ]^－^ |
| M074 |  | 425.13 (1) |  | 463.09[M+K－2H], 287.06[M－OH－2H_2_O－2CH_3_－2CO]^－^, 175.03[M－H－2CH_3_－2CO－C_8_H_9_O_2_－CO]^－^, 151.00[M－H－C_15_H_14_O_5_]^－^, 135.04[M－H－C_15_H_14_O_6_]^－^, |
| M075 | 463.12 (4) |  | 317.07[M+H－Rha]^+^,302.04[M+H－Rha－CH_3_]^+^, |  |
| M076 | 463.09 (2) |  | 287.06[M+H－GluA]^+^,153.02[M+H－GluA－C_8_H_6_O_2_]^+^ |  |
| M077 |  | 461.13 (0) |  | 299.07[M－Fructose]^－^,281.07[M－Fructose－H_2_O]^－^,239.06[M－Fructose－C_2_H_4_O_2_]^－^,137.02[M－Fructose－Glu]^－^ |
| M078 | 453.14 (8) |  | 291.09[M+H－Glu]^+^,139.04[M+H－Glu－C_8_H_7_O_3_]^+^,123.04[M+H－Glu－C_8_H_7_O_3_－CO]^+^ |  |
| M079 |  | 449.11 (5) |  | 303.05[M－Rha]^－^, 285.04[M－Rha－H_2_O]^－^, 179.00[M－H－Rha－C_7_H_7_O_2_]^－^, 151.00[M－H－Rha－C_8_H_7_O_3_]^－^RDA, 125.03[M－H－Rha－C_8_H_7_O_3_－C_2_H_2_]^－^ |
| M080 |  | 449.11 (4) |  | 287.06[M－Glu]^－^,175.00[M－H－C_8_H_8_O_2_－H_2_O－C_4_H_8_O_4_]^－^,151.00[M－Glu－C_8_H_8_O_2_]^－^ |
| M081 | 449.11 (2) | 447.09 (6) | 303.05[M+H－Rha]^+^, 153.02[M+H －Rha－C_8_H_5_O_3_]^－^, RDA, 129.06[M－C_15_H_9_O_7_－H_2_O ]^+^, | 301.04[M－Rha]^－^, 300.04[M－H－Rha]^－^, 271.03[M－H －Rha－CHO]^－^, 255.04[M－Rha－CO－H_2_O]^－^, 151.01[M－H －Rha－C_8_H_5_O_3_]^－^ |
| M082 | 449.11 (6) | 447.09 (3) | 287.06[M+H－Glu]^+^,153.02[M+H－Glu－C_8_H_5_O_2_]^+^, | 285.04[M－Glu]^－^, 284.03[M－H－Glu]^－^, 255.03[M－H－Glu－CHO]^－^, 227.04[M－H－Glu－CHO－CO]^－^ |
| M083 | 449.11 (5) | 447.09 (5) | 431.10[M－H_2_O]^+^, 413.09[M－2H_2_O]^+^, 395.08[M－3H_2_O]^+^, 353.07[M+H－－2H_2_O－C_2_H_4_O_2_]^+^, 329.07[M+H－C_4_H_8_O_4_]^+^, 299.06[M+H－Glu]^+^, 165.02[M+H－Glu－C_8_H_6_O_2_]^+^RDA, 137.02[M+H－Glu－C_8_H_6_O_2_－CO]^+^ | 357.06[M－H－C_3_H_6_O_3_]^－^,327.05[M－H－C_4_H_8_O_4_]^－^,297.04[M－H－Glu]^－^, 133.03[M－H－Glu－C_8_H_4_O_4_]^－^ |
| M084 |  | 447.09 (11) |  | 285.04[M－Glu]^－^,284.03[M－H－Glu]^－^,151.00[M－H－Glu－C_8_H_6_O_2_]^－^, 133.03[M－H－Glu－C_7_H_3_O_4_]^－^,107.01[M－H－Glu－C_7_H_3_O_4_－C_2_H_2_]^－^ |
| M085 |  | 447.09 (2) |  | 285.04[M－Glu]^－^,151.00[M－H－Glu－C_8_H_6_O_2_] |
| M086 | 431.18 (2) |  | 147.07[M+H－Rha－H_2_O－C_4_H_7_O_4_]^+^, 129.05[M+H－Rha－2H_2_O－C_4_H_7_O_4_]^+^, |  |
| M087 |  | 445.08 (4) |  | 269.05[M－GluA]^－^,113.02[M－H－GluA－C_3_HO－C_8_H_6_]^－^ |
| M088 | 445.21 (3) |  | 224.11[M－C_12_H_14_NO_3_]^+^, 194.12[M+H－C_16_H_14_NO_2_]^+^, 105.03[M－C_12_H_14_NO_3_－C_8_H_9_N]^+^, |  |
| M089 | 445.21 (2) |  | 265.14[M－C_6_H_11_O_6_]^+^,247.13[M－C_6_H_11_O_6_－H_2_O]^+^,193.09[M－C_6_H_11_O_6_－H_2_O－CO－C_2_H_2_]^+^ |  |
| M090 | 422.22 (1) | 421.21 (10) | 133.05[M－C_8_H_15_O－Glu]^+^,115.04[M－C_8_H_15_O－Glu－H_2_O]^+^, | 467.21[M+COOH]^－^, 289.16[M－Apiose]^－^, 161.04[M－H－Apiose－C_8_H_15_O]^－^ |
| M091 | 433.13 (0) |  | 455.12[M+Na]^+^, 329.08[M－C_4_H_7_O_3_]^+^, |  |
| M092 | 435.13 (4) | 433.11 (3) | 219.03[M－C_6_H_5_O －C_4_H_8_O_3_－H_2_O]^+^,195.03[M+H－C_8_H_8_O －C_4_H_8_O_4_]^+^,165.02[M+H－Glu－C_8_H_8_O]^+^, | 343.09[M－H－C_3_H_6_O_3_]^－^,313.07[M－H－C_4_H_8_O_4_]^－^,119.05[M－H－Glu－－C_8_H_4_O_4_]^－^ |
| M093 | 435.10(3) |  | 303.05[M+H－Xyl]^+^, 229.05[M+H－Xyl－2CO－H_2_O]^+^, 153.02[M+H－Xyl－C_8_H_5_O_3_]^+^,137.03[M－Xyl－C_6_H_4_O_2_－2CO]^+^ |  |
| M094 |  | 433.11 (1) |  | 271.06[M－Glu]^－^, 151.00[M－Glu－C_8_H_8_O]^－^, 119.05[M－H－Glu－C_7_H_3_O_4_]^－^ |
| M095 |  | 433.08 (1) |  | 300.03[M－H－Ara]^－^,271.03[M－H－Ara－CHO]^－^,255.03[M－Ara－CO－H_2_O]^－^,243.03[M－H－Ara－CHO－CO]^－^,151.00[M－H－Ara－C_8_H_5_O_3_]^－^ |
| M096 | 416.17(4) |  | 434.20[M+NH_4_]^+^,133.05[M－C_8_H_9_O－Glu]^+^,115.04[M－C_8_H_9_O－Glu－H_2_O]^+^, 97.03[M－C_8_H_9_O－Glu－2H_2_O]^+^, |  |
| M097 | 433.11 (0) | 431.10 (4) | 287.05[M+H－Rha]^+^,129.05[M+H－C_15_H_9_O_6_－H_2_O ]^+^, | 285.04[M－Rha]^－^,255.03[M－H－Rha－CHO]^－^,227.04[M－H－Rha－CHO－CO]^－^ |
| M098 | 433.11 (9) | 431.1 (4) | 337.07[M+H－C_2_H_4_O_2_－2H_2_O]^+^, 323.09[M－C_2_H_4_O_2_－CH_2_OH－H_2_O]^+^, 309.07[M+H－C_3_H_6_O_3_－2OH]^+^, 283.06[M+H－Glu]^+^, | 341.08[M－H－C_3_H_6_O_3_]^－^, 323.07[M－H－C_3_H_6_O_3_－H_2_O]^－^, 311.07, 283.07[M－C_4_H_8_O_3_－CO－OH]^－^ |
| M099 |  | 421.8 (6) |  | 331.06[M－H－C_3_H_6_O_3_]^－^, 301.05[M－H－C_4_H_8_O_4_]^－^, 272.04[M－Glu]^－^, 259.04[M－C_6_H_11_O_5_]^－^ |
| M100 | 403.16(7) |  | 420.19[M+NH_4_]^+^, 133.05[M－C_7_H_7_O－Glu]^+^, 115.04[M－C_7_H_7_O－Glu－H_2_O]^+^, 91.05[M－Apiose－Glu]^+^, |  |
| M101 | 419.17 (4) |  | 217.09[M+H－C_9_H_10_O_3_－H_2_O]^+^, 205.09[M+H－C_9_H_10_O_3_－CH_3_O－OH]^+^, 173.06[M－C_9_H_10_O_3_－2CH_3_O－OH ]^+^, 167.07[M+H－C_13_H_16_O_5_]^+^, 145.07[M－C_9_H_10_O_3_－2CH_3_O－OH－CO ]^+^ |  |
| M102 | 419.10 (1) | 417.08 (4) | 287.05[M+H－Ara]^+^,153.02[M+H－Ara－C_8_H_5_O_2_]^+^, | 284.03[M－H－Ara]^－^,255.03[M－H－Ara－CHO]^－^,227.03[M－H－Ara－CHO－CO]^－^,183.05[M－H－Ara－H_2_O－3CO]^－^, |
| M103 | 417.12 (9) |  | 307.10[M+H－C_3_H_6_O_2_－2H_2_O]^+^, 297.08[M+H－C_4_H_8_O_4_]^+^, 267.07[M+H－C_5_H_10_O_5_]^+^ |  |
| M104 | 389.22(2) |  | 406.24[M+NH_4_]^+^, 209.16[M－C_6_H_11_O_6_]^+^, 191.14[M－C_6_H_11_O_6_－H_2_O]^+^, 153.09[M－Glu－C_3_H_5_O－CH_3_]^+^, 111.08[M－Glu－C_5_H_7_O_2_－CH_3_]^+^ |  |
| M105 | 389.18(2) |  | 406.21[M+NH_4_]^+^, 227.13[M+H－Glu]^+^, 209.12[M+H－Glu－H_2_O]^+^, 191.11[M+H－Glu－2H_2_O]^+^, 167.11[M+H－Glu－CH_2_COOH]^+^, 149.09[M+H－Glu－CH_2_COOH－H_2_O]^+^, 131.09[M+H－Glu－CH_2_COOH－2H_2_O]^+^ |  |
| M106 | 368.11 (3) |  | 391.10[M+Na]^+^,215.05[M－C_4_H_6_O_3_－ 3OH]^+^, 199.04[M－C_4_H_6_O_3_－CH_3_O－2H_2_O]^+^, 177.06[M－C_7_H_11_O_6_]^+^, 145.03[M－C_7_H_11_O_6_－CH_3_O－OH]^+^ |  |
| M107 |  | 385.11 (4) |  | 223.06[M－Glu]^－^,205.05[M－Glu－H_2_O]^－^,190.03[M－Glu－H_2_O－CH_3_]^－^,175.00[M－Glu－H_2_O－CH_3_]^－^ |
| M108 | 372.14(2) |  | 390.18[M+NH_4_]^+^, 193.09[M－C_6_H_11_O_6_]^+^, 161.06[M－C_6_H_11_O_6_－CH_3_O－OH]^+^, 133.07[M－C_6_H_11_O_6_－CH_3_O－OH－CO]^+^,105.07[M－C_6_H_11_O_6_－CH_3_O－OH－2CO]^+^ |  |
| M109 | 389.22 (5) |  | 209.15[M－C_6_H_11_O_6_]^+^,191.14[M－C_6_H_11_O_6_－H_2_O]^+^,149.10[M－C_6_H_11_O_6_－H_2_O－CH ( CH3)_2_]^+^,125.10[M－C_6_H_11_O_6_－CHCH_3_－OH－CHCCH_3_]^+^, |  |
| M110 | 377.15 (2) |  | 243.09[M+H－C_5_H_11_O_4_]^+^, 198.07[M－C_5_H_11_O_4_－CONH]^+^, 172.09[M－C_5_H_11_O_4_－CNCONH]^+^, |  |
| M111 | 375.24 (0) |  | 213.19[M+H－Glu]^+^, 195.17[M+H－Glu－H_2_O]^+^, 177.16[M+H－Glu－2H_2_O]^+^, 121.10[M－Glu－C_4_H_8_O－H_2_O]^+^, 107.09[M+H－Glu－C_4_H_8_O－H_2_O－CH_3_]^+^ |  |
| M112 | 373.22 (2) |  | 211.17[M+H－Glu]^+^, 193.16[M+H－Glu－H_2_O]^+^, 175.15[M+H－Glu－2H_2_O]^+^, 135.12[M+H－Glu－2H_2_O－CHCCH_3_]^+^, 109.10[M+H－Glu－C_4_H_8_O]^+^ |  |
| M113 | 356.19 (2) |  | 310.12[M+H－CH_3_O－CH_3_]^+^, 294.13[M+H－2CH_3_O]^+^, 279.10[M+H－2CH_3_O－CH_3_]^+^, 251.11[M+H－2CH_3_O－CH_3_－CO]^+^, |  |
| M114 | 356.19 (5) |  | 192.10[M－4CH_3_O－CCH CH_2_]^+^ |  |
| M115 | 355.10 (8) | 353.09 (1) | 163.04[M－C_7_H_11_O_6_]^+^, 145.02[M－C_7_H_11_O_6_－H_2_O]^+^, 135.04[M－C_7_H_11_O_6_－CO]^+^, 117.04[M－C_7_H_11_O_6_－2CO]^+^ | 191.06[M－C_9_H_7_O_3_]^－^,179.03[M－C_7_H_11_O_5_]^－^,173.05[M－C_9_H_7_O_3_－H_2_O]^－^ |
| M116 | 355.10 (2) |  | 319.08[M+H－2H_2_O]^+^, 235.06[M+H－C_4_H_8_O_4_]^+^, 205.05[M+H－Glu]^+^, |  |
| M117 | 347.13 (2) |  | 185.08[M+H－Glu]^+^, 153.06[M+H－Glu－CH_3_OH]^+^, 139.04[M+H－Glu－CO－H_2_O]^+^, 125.06[M+H－Glu－CH_3_OH－CO]^+^, 110.04[M+H－Glu－CH_3_OH－CH_3_－CO]^+^ |  |
| M118 |  | 341.10 (2) |  | 309.08[M－H－CH_3_OH]^－^, 265.08[M－H－CH_3_OH－CO_2_]^－^, 237.09[M－H－ CH_3_OH－CO_2_－CO]^－^, 143.05[M－H－ CH_3_OH－CO－COOH－C_6_H_5_O]^－^, 121.03[M－ C_9_H_8_O_3_－CH_3_O－C_2_H_2_]^－^ |
| M119 | 359.15(2) |  | 341.14[M+H－H_2_O]^+^, 323.13[M+H－2H_2_O]^+^, 291.10[M+H－2H_2_O－CH_3_OH]^+^, 187.07[M+H－2H_2_O－CH_3_OH－C_7_H_4_O]^+^, 137.06[M+H－C_12_H_14_O_4_]^+^ |  |
| M120 | 340.15 (8) |  | 176.07[M+H－C_10_H_12_O_2_]^+^,149.06[M－C_10_H_12_O_2_－CO]^+^ |  |
| M121 | 349.17(0) |  | 331.15[M+H－H_2_O]^+^, 285.12[M+H－2H_2_O－CH_3_CH]^+^, 253.12[M－2H_2_O－CH_3_COOH]^+^, 195.08[M－3CH_3_－CH_3_COOH－H_2_O－CHOH]^+^, 151.08[M－2CH_3_－2CH_3_COOH－H_2_O－CHO]^+^, 137.06[M+H－3CH_3_－2CH_3_COOH－H_2_O－CO]^+^, |  |
| M122 | 330.17 (5) |  | 299.15[M+H－CH_3_O]^+^, 192.10[M－C_8_H_9_O_2_]^+^, 175.09[M+H－H_2_O－C_8_H_9_O_2_]^+^, 137.06[M－C_11_H_14_NO_2_]^+^ |  |
| M123 | 317.07 (7) | 315.05 (9) | 302.04[M+H－CH_3_]^+^, 285.04[M+H－CH_3_OH]^+^, 274.05[M+H－CH_3_CO]^+^, 245.05[M+H－CHO－CH_3_CO]^+^, 229.05[M－CO_2_－CH_3_CO]^+^, 217.05[M－2CO－CH_3_CO]^+^, 153.02[M+H－C_9_H_8_O_3_]^+^ | 300.03[M－H－CH_3_]^－^, 271.02[M－H－CH_3_－CHO]^－^, 183.01, 151.00[M－H －C_9_H_8_O_3_]^－^ |
| M124 | 314.14 (6) |  | 177.06[M－C_8_H_10_NO]^+^, 145.03[M－C_8_H_10_NO－CH_3_OH]^+^, 121.07[M－C_10_H_10_NO3]^+^ |  |
| M125 | 327.11(1) |  | 309.10[M+H－H_2_O]^+^, 147.04[M－C_6_H_11_O_6_]^+^, 119.05[M－C_6_H_11_O_6_－CO]^+^, 91.05[M－C_6_H_11_O_6_－2CO]^+^ |  |
| M126 | 308.09 (3) |  | 231.04[M+H－HCOOH－NH_2_CH_3_]^+^, 177.03[M－C_5_H_8_NO_3_]^+^, 130.05[M+H－SH－C_5_H_9_N_2_O_3_]^+^, 84.04[M+H－SH－C_5_H_9_N_2_O_3_－HCOOH]^+^ |  |
| M127 | 305.06 (3) | 303.05 (3) | 259.06[M+H－H_2_O－CO]^+^, 231.07[M+H－H_2_O－2CO]^+^, 153.02[M+H－ C_8_H_8_O_3_]^+^, 149.02[M－C_6_H_5_O_2_－H_2_O－CO ]^+^, 123.04[M－C_8_H_8_O_3_－CHO]^+^ | 285.04[M－H－H_2_O]^－^, 217.05[M－H－CO－2CHO]^－^, 175.04[M－2CHO－ C_3_H_3_O_2_]^－^, 125.02[M－ C_3_H_2_O_2_－C_6_H_5_O_2_]^－^ |
| M128 | 303.05 (1) | 301.03 (6) | 229.05[M+H－H_2_O－2CO]^+^, 201.06[M+H－H_2_O－3CO]^+^, 153.02[M+H－C_8_H_6_O_3_]^+^RDA, 137.02[M－C_6_H_4_O_2_－CHO－CO]^+^ | 179.00[M－H－C_7_H_6_O_2_]^－^, 151.00[M－H－C_8_H_6_O_3_]^－^RDA, 121.03[M－H－C_8_H_4_O_5_]^－^, 107.01[M－H－C_9_H_6_O_5_]^－^ |
| M129 | 297.32 (3) |  | 281.28[M－CH_3_]^+^, 265.29[M－CH_2_OH]^+^, 249.26[M+H－2CH_3_－H_2_O]^+^ |  |
| M130 | 311.22(8) |  | 293.21[M+H－H_2_O]^+^, 145.10[M－CO_2_－C_9_H_13_]^+^, 105.07[M+H－2H_2_O－C_5_H_9_－C_5_H_9_O_2_]^+^, 81.07[M+H－H_2_O_2_－C_7_H_11_－C_5_H_9_O_2_]^+^, 91.05[M+H－2H_2_O－C_5_H_9_－C_6_H_11_O_2_]^+^, 81.07[M+H－H_2_O_2_－C_7_H_11_－C_5_H_9_O_2_]^+^, 67.05[M+H－H_2_O_2_－C_7_H_11_－C_6_H_11_O_2_]^+^ |  |
| M131 | 291.09 (2) | 289.07 (6) | 147.04[M+H－C_6_H_4_O_2_－2H_2_O]^+^, 139.04[M+H－C_8_H_8_O_3_]^+^, 123.04[M－CHO－C_7_H_6_O_3_]^+^ | 245.08[M－CO－OH]^－^, 203.07[M－H－2CO－CHOH]^－^, 151.04[M－H－C_7_H_6_O_3_]^－^, 137.02[M－H－C_8_H_8_O_3_]^－^, 123.05[M－H－CO－C_7_H_6_O_3_]^－^, 109.03[M－H－C_8_H_8_O_3_－CO]^－^ |
| M132 | 289.07 (4) |  | 243.06[M+H－CO－H_2_O]^+^, 215.07[M+H－2CO－H_2_O]^+^, 153.02[M+H－C_8_H_8_O_2_]^+^, 149.02[M+H－C_6_H_4_O_2_－CH_3_OH]^+^, 107.05[M+H－C_7_H_4_O_4_－CHOH]^+^ |  |
| M133 | 287.05 (1) | 285.04 (10) | 153.02[M+H－C_8_H_6_O_2_]^+^RDA,135.04[M+H－C_7_H_4_O_4_]^+^RDA | 151.00[M－H－C_8_H_6_O_2_]^－^,133.03[M－H－C_7_H_4_O_4_]^－^RDA,107.01[M－H－C_7_H_4_O_4_－C_2_H_2_]^－^ |
| M134 | 287.06 (5) | 285.04 (4) | 213.06[M+H－2CO－H_2_O]^+^,153.02[M+H－C_8_H_6_O_2_]^+^,121.03[M－C_6_H_4_O_2_－CO－CHO] | 239.03[M－H－CO－H_2_O]^－^, 227.03[M－H－2CHO]^－^, 211.04[M－H－2CO－H_2_O]^－^, 187.03[M－C_3_H_3_O_2_－CO]^－^, 159.04[M－C_3_H_3_O_2_－2CO]^－^, 143.05[M－H－4CO－CHOH]^－^ |
| M135 | 285.08 (0) |  | 270.05[M+H－CH_3_]^+^,168.00[M+H－CH_3_－C_8_H_6_]^+^ |  |
| M136 | 284.10 (6) |  | 152.06[M+H－Ribose]^+^,135.03[M+H－Ribose－NH_3_]^+^,110.04[M+H－Ribose－NH_2_CN]^+^ |  |
| M137 | 294.23 (1) |  | 277.22[M+H－H_2_O]^+^, 149.13[M+H－H_2_O－CH_2_COOH－C_5_H_9_]^+^, 135.12[M+H－H_2_O－CH_2_CH_2_COOH－C_5_H_9_]^+^, 121.10[M－C_9_H_17_O_3_]^+^, 107.09[M+H－CH_3_－C_9_H_17_O_3_]^+^, |  |
| M138 | 277.14 (7) |  | 259.13[M+H－H_2_O]^+^, 213.12[M+H－H_2_O－HCOOH]^+^, 130.09[M－C_5_H_8_NO_4_]^+^, 84.08[M－C_5_H_8_NO_4_－HCOOH]^+^ |  |
| M139 | 293.21 (4) |  | 275.20[M+H－H_2_O]^+^, 147.12[M+H－H_2_O－C_3_H_5_O_2_－C_4_H_7_]^+^, 133.10[M+H－H_2_O－C_4_H_7_O_2_－C_4_H_7_]^+^, 119.09[M+H－H_2_O－C_5_H_9_O_2_－C_4_H_7_]^+^, 105.07[M+H－H_2_O－C_6_H_11_O_2_－C_4_H_7_]^+^, 91.05[M+H－H_2_O－C_7_H_13_O_2_－C_4_H_7_]^+^ |  |
| M140 | 273.08 (5) | 271.06 (6) | 153.02[M+H－C_8_H_8_O]^+^, 147.04[M+H－C_6_H_4_O_2_－H_2_O]^+^, 119.05[M+H－C_6_H_4_O_2_－H_2_O－CO]^+^ | 151.00[M－H－C_8_H_8_O]^+^, 119.05[M－H－C_7_H_4_O_4_]^+^ |
| M141 | 271.06 (1) | 269.04 (1) | 153.02[M+H－C_8_H_8_O]^+^,119.05[M+H－C_7_H_4_O_4_]^+^, | 225.06[M－CO－OH]^－^, 151.00[M－H－C_8_H_6_O]^+^, 117.04[M－H－C_7_H_4_O_4_]^+^ |
| M142 | 268.10 (8) |  | 222.11[M－CHOH－H_2_O]^+^, 136.06 [M+H－Ribose]^+^ |  |
| M143 | 291.23 (5) |  | 255.21[M+H－2H_2_O]^+^, 129.10[M+H－2H_2_O－2CH_3_－C_7_H_11_]^+^, 105.06[M+H－2H_2_O－C_2_H_4_－C_9_H_14_]^+^, 91.06[M－2H_2_O－CH_3_－C_11_H_16_]^+^, 79.06[M+H－2H_2_O－C_13_H_20_]^+^, |  |
| M144 |  | 253.05 (8) |  | 209.06[M－CO－OH]^－^, 145.03[M－H－C_6_H_4_O_2_]^+^ RDA裂解, 143.05[M－2OH－C_6_H_5_]^+^ |
| M145 | 244.10 (4) | 242.08 (3) | 112.10[M+H－Ara]^+^ | 110.04[M－Ara]^－^,109.03[M－H－Ara]^－^, 81.03[M－H－Ara－CO]^－^ |
| M146 | 227.13 (8) |  | 209.12[M+H－H_2_O]^+^, 191.11[M+H－2H_2_O]^+^, 149.10[M－H_2_O－CH_2_COOH]^+^, 131.09[M－2H_2_O－CH_2_COOH]^+^ |  |
| M147 | 225.08 (7) | 223.06 (8) | 207.07[M+H－H_2_O]^+^, 175.04[M－ H_2_O－CH_3_O]^+^, 147.05[M－ H_2_O－CH_3_－CO_2_]^+^, 119.05[M－ H_2_O－CH_3_－CO_2_－CO]^+^ | 208.04[M－H－CH_3_]^－^, 193.02[M－H－2CH_3_]^－^, 164.05[M－H－CH_3_－CO_2_]^－^, 149.03[M－H－2CH_3_－CO_2_]^－^, 121.03[M－H－2CH_3_－CO_2_－CO]^－^ |
| M148 | 223.06 (8) |  | 208.04[M+H－CH_3_]^+^, 190.03[M+H－CH_3_－H_2_O]^+^, 162.03[M+H－CH_3_－H_2_O－CO]^+^,134.04[M+H－CH_3_－H_2_O－2CO]^+^, 105.04[M+H－CH_3_－H_2_O－2CO－CHO]^+^ |  |
| M149 | 220.12 (5) | 218.10 (4) | 142.09[M－CH_3_－CO_2_－H_2_O]^+^, 124.08[M－CH_3_－CO_2_－2H_2_O]^+^, 98.02[M－H_2_O－ C_5_H_11_O_2_]^+^, 90.05[M+H － C_6_H_10_O_3_]^+^, 72.05[M－CO_2_－C_5_H_11_O_2_]^+^ | 146.08[M－C_3_H_5_O_2_]^－^, 88.04[M－C_6_H_11_O_3_]^－^ |
| M150 | 213.08 (1) |  | 149.06[M+H－H_2_O－HCOOH]^+^, 135.08[M+H－2OH－CO_2_]^+^, 125.06[M－CH_3_－CO－CO_2_]^+^, 121.07[M+H－H_2_O－HCOOH－CO]^+^, 107.05[M－CH_3_－CO－CO_2_－H_2_O]^+^, 93.00[M+H－H_2_O－HCOOH－2CO]^+^, 79.05[M－CH_3_－2CO－CO_2_－H_2_O]^+^ |  |
| M151 |  | 209.03 (6) |  | 191.02[M－H－H_2_O]^－^, 133.01[M－H_2_O－CH_2_COOH]^－^, 89.02[M－H－CO_2_－HOCH_2_COOH]^－^, 85.03[M－H－2H_2_O－2CO_2_]^－^ |
| M152 |  | 206.08 (3) |  | 164.07[M－CH_3_CO]^－^, 147.05[M－H－CH_3_CONH_2_]^－^, 103.05[M－H－CO_2_－CH_3_CONH_2_]^－^, 91.05[M－C_4_H_6_NO_3_]^－^ |
| M153 | 341.09 (1) |  | 207.07[M－C_4_H_5_O_5_]^+^, 175.04[M－C_4_H_5_O_5_－CH_3_OH]^+^, 147.04[M－C_4_H_5_O_5_－CH_3_OH－CO]^+^, 119.05[M－C_4_H_5_O_5_－CH_3_OH－2CO]^+^ |  |
| M154 | 205.10 (4) | 203.08 (3) | 143.07[M－NH_3_－CO_2_]^+^, 118.07[M+H－C_3_H_5_NO_2_]^+^, 91.04[M+H－C_5_H_8_NO_2_]^+^ | 143.07[M－NH_3_－CO_2_]^－^, 142.07[M－H－NH_3_－CO_2_]^－^, 116.05[M－C_3_H_6_NO_2_]^－^ |
| M155 | 195.06 (5) |  | 177.05[M+H－H_2_O]^+^, 149.06[M+H－H_2_O－CO]^+^, 145.03[M+H－CH_3_OH－H_2_O]^+^, 134.04[M+H－H_2_O－CO－CH_3_]^+^, 117.03[M+H－H_2_O－CH_3_－COOH]^+^, 89.04[M+H－CH_3_－CO－H_2_O－COOH]^+^ |  |
| M156 |  | 195.05 (2) |  | 129.02[M－CH_2_OH－2H_2_O]^－^, 99.01[M－CH_2_OH－2H_2_O]^－^, 75.00[M－C_4_H_9_O_4_]^－^ |
| M157 | 193.09 (5) |  | 161.06[M+H－CH_3_O]^+^, 133.06[M+H－CH_3_O－CO]^+^, 115.05[M+H－CH_3_OH－CH_2_O_2_ ]^+^, 105.07[M+H－CH_3_O－CO]^+^ |  |
| M158 | 193.05 (0) |  | 178.03[M+H－CH_3_]^+^, 150.04[M+H－CH_3_－CO]^+^, 137.06[M+H－2CO]^+^, 133.03[M+H－CH_4_－CO_2_]^+^, 122.04[M+H－CH_3_－2CO]^+^ |  |
| M159 |  | 191.02 (10) |  | 111.01[M－H－CO_2_－2H_2_O]^－^, 87.01[M－H－CO_2_－CH_3_COOH]^－^, 85.03[M－H－2CO_2_－H_2_O]^－^ |
| M160 | 219.11(2) |  | 188.07[M+H－CH_3_NH_2_]^+^, 143.07[M+H－CH_3_NH_2_－COOH]^+^, 118.06[M+H－C_4_H_7_NO_2_]^+^, 115.05[M+H－CH_3_－ C_3_H_7_NO_2_]^+^ |  |
| M161 |  | 187.10 (5) |  | 125.10[M－H－CO_2_－H_2_O]^－^ |
| M162 |  | 181.07 (6) |  | 101.02[M－H－H_2_O－2CH_2_OH]^－^, 89.02[M－H－C_2_H_5_O_2_－CH_2_OH]^－^, 71.01[M－H－H_2_O－C_2_H_5_O_2_－CH_2_OH]^－^, 59.01[M－H－C_3_H_7_O_3_－CH_2_OH]^－^ |
| M163 | 182.08 (5) |  | 135.07[M－HCOOH]^+^, 122.06[M+H－H_2_O－C_2_H_2_O]^+^, 118.07[M+H－H_2_O－HCOOH]^+^, 95.07[M+H－HCOOH－C_2_HO]^+^, 91.05[M+H－HCOOH－NH_3_－CO]^+^, |  |
| M164 | 181.05 (5) |  | 163.04[M+H－H_2_O]^+^, 145.03[M+H－2H_2_O]^+^, 135.04[M+H－HCOOH]^+^, 117.03[M+H－HCOOH－H_2_O]^+^, 89.04[M+H－HCOOH－H_2_O－CO]^+^, |  |
| M165 | 175.12 (5) |  | 130.10[M+H－COOH]^+^, 116.07[M+H－(NH_2_)_2_NH]^+^, 70.07[M+H－(NH_2_)_2_NH－HCOOH]^+^, 60.06[M＋H－C_5_H_10_NO_2_]^+^ |  |
| M166 |  | 194.06 (0) |  | 149.06[M－H－CO_2_]^－^, 145.03[M－CH_3_O－H_2_O]^－^, 134.04[M－H－CO_2_－CH_3_]^－^, 117.03[M－CO_2_－CH_3_－H_2_O]^－^, 89.04[M－CO_2_－CH_3_O－CHO]^－^ |
| M167 |  | 177.02 (1) |  | 133.03[M－H－CO_2_]^－^, 105.04[M－H－CO_2_－CO]^－^, 89.04[M－H－CO_2_－CO_2_]^－^ |
| M168 | 449.11 (6) |  | 317.06[M+H－Xyl]^+^, 302.04[M+H－Xyl－CH_3_]^+^, 274.05[M+H－Xyl－CH_3_－CO]^+^ |  |
