# Supplementary material for: Integration of molecular networking and fingerprint analysis for studying constituents in Microctis Folium
Source: PLoS One. 2020 Jul 7;15(7):e0235533. doi: 10.1371/journal.pone.0235533 (PMC7340309; doi:10.1371/journal.pone.0235533)
Supplement: S4 Table — (DOCX) [file pone.0235533.s009.docx]

S4 Table Molecular networking families’ information

| Family Name. | Node  Count | Identified Compound Number |
| --- | --- | --- |
| Pos-01 | 100 | M004, M005, M007, M008, M009, M010,M013, M014, M018, M019, M020, M021, M025, M028, M032, M038, M065, M068, M072, M075, M081, M082, M093, M097, M102, M168 |
| Pos-02 | 100 | M031, M033, M041, M042, M049, M050, M051,M083, M098, M103, M116 |
| Pos-03 | 87 | M143 |
| Pos-04 | 54 | M043 |
| Pos-05 | 36 | M131 |
| Pos-06 | 33 | M106, M115, M124 |
| Pos-07 | 25 | M138 |
| Pos-08 | 24 | M086, M090, M096, M100, |
| Pos-09 | 23 | M147, M153, M155, M163, M164, |
| Pos-10 | 19 | M123, M128, M134, |
| Pos-11 | 16 | M108 |
| Pos-12 | 13 | M043, M044 |
| Pos-13 | 13 | M111, M057, M112 |
| Pos-14 | 12 | M091 |
| Pos-15 | 10 | M052 |
| Pos-16 | 8 | M127 |
| Pos-17 | 7 | M132 |
| Pos-18 | 6 | M027 |
| Pos-19 | 6 | M053 |
| Pos-20 | 6 | M133, M141 |
| Pos-21 | 6 | M140 |
| Pos-22 | 6 | M110 |
| Pos-23 | 6 | M069 |
| Pos-24 | 5 | M119 |
| Pos-25 | 5 | M104 |
| Pos-26 | 5 | M131 |
| Pos-27 | 4 | M056 |
| Pos-28 | 4 | M127 |
| Pos-29 | 3 | M132 |
| Pos-30 | 3 | M027 |
| Pos-31 | 3 | M053 |
| Pos-32 | 3 | M133, M141 |
| Pos-33 | 3 | M140 |
| Pos-34 | 3 | M110 |
| Pos-35 | 2 | M069 |
| Pos-36 | 2 | M119 |
| Pos-37 | 2 | M104 |
| Pos-38 | 2 | M131 |
| Pos-39 | 2 | M056 |
| Pos-40 | 2 | M127 |
| Neg-01 | 84 | M043 |
| Neg-02 | 76 | M055, M057, M058, M059, M090 |
| Neg-03 | 62 | M003, M018, M002, |
| Neg-04 | 61 | M037, M041, M049, M030 |
| Neg-05 | 55 | M052 |
| Neg-06 | 52 | M029 |
| Neg-07 | 51 | M060, M083, M098, M092 |
| Neg-08 | 34 | M067 |
| Neg-09 | 28 | M066,M018 |
| Neg-10 | 24 | M016, M023, M038, M071, M081, M095 |
| Neg-11 | 23 | M001 |
| Neg-12 | 23 | M029, M039, M040, M074 |
| Neg-13 | 20 | M131, M133, M140, M141 |
| Neg-14 | 16 | M107 |
| Neg-15 | 14 | M011, M012 |
| Neg-16 | 13 | M058 |
| Neg-17 | 10 | M054, M115 |
| Neg-18 | 10 | M049 |
| Neg-19 | 9 | M065 |
| Neg-20 | 8 | M077 |
| Neg-21 | 7 | M043 |
| Neg-22 | 6 | M156 |
| Neg-23 | 5 | M082, M097, M102 |
| Neg-24 | 5 | M034 |
| Neg-25 | 4 | M023 |
| Neg-26 | 4 | M128 |
| Neg-27 | 4 | M057 |
| Neg-28 | 4 | M149 |
| Neg-29 | 3 | M162 |
| Neg-31 | 3 | M087 |
| Neg-32 | 3 | M166 |
| Neg-33 | 3 | M131 |
| Neg-34 | 3 | M010 |
| Neg-35 | 3 | M058 |
| Neg-36 | 3 | M118 |
| Neg-37 | 3 | M057 |
| Neg-38 | 3 | M027 |
| Neg-39 | 3 | M127 |
| Neg-40 | 3 | M134 |
| Neg-41 | 3 | M123 |
| Neg-42 | 2 | M048 |
| Neg-43 | 2 | M017 |
| Neg-44 | 2 | M147 |
| Neg-45 | 2 | M045 |
| Neg-46 | 2 | M043 |
| Neg-47 | 2 | M070 |
| Neg-48 | 2 | M091 |
| Neg-49 | 2 | M018 |
| Neg-50 | 2 | M005 |
| Neg-51 | 2 | M009 |
| Neg-52 | 2 | M079 |
| Neg-53 | 2 | M152 |
