# Supplementary material for: Integration of molecular networking and fingerprint analysis for studying constituents in Microctis Folium
Source: PLoS One. 2020 Jul 7;15(7):e0235533. doi: 10.1371/journal.pone.0235533 (PMC7340309; doi:10.1371/journal.pone.0235533)
Supplement: Attachment — Submitted filename: Response to reviewer 20200314.docx [file pone.0235533.s010.docx]

**Comments to the Author**

1. Is the manuscript technically sound, and do the data support the conclusions?

The manuscript must describe a technically sound piece of scientific research with data that supports the conclusions. Experiments must have been conducted rigorously, with appropriate controls, replication, and sample sizes. The conclusions must be drawn appropriately based on the data presented.

Reviewer #1: Yes

**Response:**

**Thanks for your comment and approval.**

2. Has the statistical analysis been performed appropriately and rigorously?
Reviewer #1: Yes

**Response:**

**Thanks for your comment and approval.**

3. Have the authors made all data underlying the findings in their manuscript fully available?

The [PLOS Data policy](http://www.plosone.org/static/policies.action#sharing) requires authors to make all data underlying the findings described in their manuscript fully available without restriction, with rare exception (please refer to the Data Availability Statement in the manuscript PDF file). The data should be provided as part of the manuscript or its supporting information, or deposited to a public repository. For example, in addition to summary statistics, the data points behind means, medians and variance measures should be available. If there are restrictions on publicly sharing data—e.g. participant privacy or use of data from a third party—those must be specified.

Reviewer #1: Yes

**Response:**

**Thanks for your comment and approval.**

4. Is the manuscript presented in an intelligible fashion and written in standard English?

PLOS ONE does not copyedit accepted manuscripts, so the language in submitted articles must be clear, correct, and unambiguous. Any typographical or grammatical errors should be corrected at revision, so please note any specific errors here.

Reviewer #1: Yes

**Response:**

**Thanks for your comment and approval.**

5. Review Comments to the Author
Please use the space provided to explain your answers to the questions above. You may also include additional comments for the author, including concerns about dual publication, research ethics, or publication ethics. (Please upload your review as an attachment if it exceeds 20,000 characters)

Reviewer #1:

1. Author identified 165 constituents in source material MF using MN methodology, Is it exact identification without overlapping in the data used.

**Response:**

**Yes. The identified constituent detailed information was shown in S1 Fig and S2 and S3 Tables, including name, molecular structure, chemical formula, rentention time, observed exact mass, mass error, and main fragment ions. In line 220-231, our results were compared with the reported results in literature about Microctis Folium. All information presented exact identification results without overlapping in the data used.**

2. Author extracted MF with 70% methanol and used cured extract itself. However, it would be better if the extract was fractionated with different solvents to reduce complexity and to say exactly which portion and constituents are responsible for its medicinal use.

**Response:**

**In line 48-58, MF showed the effects of aiding the digestive system, alleviating diarrhoea, and treating symptoms of fever caused by the common cold when it is decocted with hot water. So, in this study, we preferred to extract hydrophilic constituents in MF, which were extracted with 50%, 70% and 100% methanol.**

3. Author has collected the source material from three districts of Guangdong province in three seasons to assess the geographical and seasonal influence on constituents. But these districts may not vary much geographically. It would be better if author collect MF from varied places of china or world.

**Response:**

**Initially, we tried to collect MF from more districts. However, we found MF was hardly available from many places of world by local herbal markets or online shopping system. Furthermore, some batches of MF failed to provide detailed location information, including latitude, longitude, and altitude, and couldn’t be used in this study, which hindered MF collection from varied places of china or world.**

6. PLOS authors have the option to publish the peer review history of their article ([what does this mean?](https://journals.plos.org/plosone/s/editorial-and-peer-review-process#loc-peer-review-history)). If published, this will include your full peer review and any attached files.

If you choose “no”, your identity will remain anonymous but your review may still be made public.

**Do you want your identity to be public for this peer review?** For information about this choice, including consent withdrawal, please see our [Privacy Policy](https://www.plos.org/privacy-policy).

Reviewer #1: No

**Response:**

**We respect reviewer’s choice.**

[NOTE: If reviewer comments were submitted as an attachment file, they will be attached to this email and accessible via the submission site. Please log into your account, locate the manuscript record, and check for the action link "View Attachments". If this link does not appear, there are no attachment files to be viewed.]

While revising your submission, please upload your figure files to the Preflight Analysis and Conversion Engine (PACE) digital diagnostic tool, <https://pacev2.apexcovantage.com/>. PACE helps ensure that figures meet PLOS requirements. To use PACE, you must first register as a user. Registration is free. Then, login and navigate to the UPLOAD tab, where you will find detailed instructions on how to use the tool. If you encounter any issues or have any questions when using PACE, please email us at [figures@plos.org](mailto:figures@plos.org). Please note that Supporting Information files do not need this step.

Dear Reviewer,

Thank you for your careful comments concerning our manuscript entitled “Integration of molecular networking and fingerprint analysis for studying constituents in Microctis Folium” [PONE-D-19-30659].

We have studied comments carefully and responded to reviewer which we hope meet with approval. Revised portion are marked using the "Track Changes" function in Microsoft Word and the responses are followed the reviewer’s comments.

We appreciate your kind advice and careful work. If anything is not clear or in case of whatever question in the manuscript, please do not hesitate to contact us.
