# Supplementary material for: Integration of molecular networking and fingerprint analysis for studying constituents in Microctis Folium
Source: PLoS One. 2020 Jul 7;15(7):e0235533. doi: 10.1371/journal.pone.0235533 (PMC7340309; doi:10.1371/journal.pone.0235533)
Supplement: Attachment — Submitted filename: Response.docx [file pone.0235533.s011.docx]

PONE-D-19-30659R1
Integration of molecular networking and fingerprint analysis for studying constituents in Microctis Folium
PLOS ONE

Dear Dr. Bai,

Thank you for submitting your manuscript to PLOS ONE. After careful consideration, we feel that it has merit but does not fully meet PLOS ONE’s publication criteria as it currently stands. Therefore, we invite you to submit a revised version of the manuscript that addresses the points raised during the review process.

The internal editorial staff has assessed your submission, and we have concerns about the grammar, usage, and overall readability of the manuscript. We therefore request that you revise the text to fix the grammatical errors and improve the overall readability of the text in order to meet PLOS ONE publication criteria requiring that the language in submitted articles must be clear, correct, and unambiguous. We suggest you have a fluent, preferably native, English-language speaker thoroughly copyedit your manuscript for language usage, spelling, and grammar. If you do not know anyone who can do this, you may wish to consider employing a professional scientific editing service. Whilst you may use any professional scientific editing service of your choice, PLOS has partnered with both American Journal Experts (AJE) to provide discounted services to PLOS authors. AJE has extensive experience helping authors meet PLOS guidelines and can provide language editing, translation, manuscript formatting, and figure formatting to ensure your manuscript meets our submission guidelines. If the PLOS editorial team finds any language issues in text that AJE has edited, AJE will re-edit the text for free. To take advantage of this special partnership, use the following link: https://www.aje.com/go/plos/. Please note that having the manuscript copyedited by AJE or any other editing services does not guarantee acceptance for publication.

Please note that PLOS ONE does not copyedit accepted manuscripts and that one of our criteria for publication is that articles must be presented in an intelligible fashion and written in clear, correct, and unambiguous English (http://www.plosone.org/static/publication#language).

We would appreciate receiving your revised manuscript by Jul 04 2020 11:59PM. When you are ready to submit your revision, log on to https://www.editorialmanager.com/pone/ and select the 'Submissions Needing Revision' folder to locate your manuscript file.

If you would like to make changes to your financial disclosure, please include your updated statement in your cover letter.

To enhance the reproducibility of your results, we recommend that if applicable you deposit your laboratory protocols in protocols.io, where a protocol can be assigned its own identifier (DOI) such that it can be cited independently in the future. For instructions see: <http://journals.plos.org/plosone/s/submission-guidelines#loc-laboratory-protocols>

Please include the following items when submitting your revised manuscript:

- A rebuttal letter that responds to each point raised by the academic editor and reviewer(s). This letter should be uploaded as separate file and labeled 'Response to Reviewers'.
- A marked-up copy of your manuscript that highlights changes made to the original version. This file should be uploaded as separate file and labeled 'Revised Manuscript with Track Changes'.
- An unmarked version of your revised paper without tracked changes. This file should be uploaded as separate file and labeled 'Manuscript'.

Please note while forming your response, if your article is accepted, you may have the opportunity to make the peer review history publicly available. The record will include editor decision letters (with reviews) and your responses to reviewer comments. If eligible, we will contact you to opt in or out.

We look forward to receiving your revised manuscript.

Kind regards,

Tommaso Lomonaco, Ph.D
Academic Editor
PLOS ONE

and

Vanessa Carels

Staff Editor

PLOS ONE
[Note: HTML markup is below. Please do not edit.]

Dear Dr.Tommaso Lomonaco,

Thanks for your careful comments concerning our manuscript entitled “Integration of molecular networking and fingerprint analysis for studying constituents in Microctis Folium” (PONE-D-19-30659R1).

We have submitted the manuscript to American Journal Experts (AJE) for English editing. Revised portion are marked using the "Track Changes" function in Microsoft Word and the responses are followed the reviewer’s comments.

We appreciate your kind advice and careful work. If anything is not clear or in case of whatever question in the manuscript, please do not hesitate to contact us.

Best wishes

Yang Bai

Reviewers' comments:

Reviewer's Responses to Questions
**Comments to the Author**
1. If the authors have adequately addressed your comments raised in a previous round of review and you feel that this manuscript is now acceptable for publication, you may indicate that here to bypass the “Comments to the Author” section, enter your conflict of interest statement in the “Confidential to Editor” section, and submit your "Accept" recommendation.

Reviewer #1: All comments have been addressed

**Response:**

**Thanks for your comment and approval.**

2. Is the manuscript technically sound, and do the data support the conclusions?
The manuscript must describe a technically sound piece of scientific research with data that supports the conclusions. Experiments must have been conducted rigorously, with appropriate controls, replication, and sample sizes. The conclusions must be drawn appropriately based on the data presented.

Reviewer #1: Yes

**Response:**

**Thanks for your comment and approval.**

3. Has the statistical analysis been performed appropriately and rigorously?

Reviewer #1: Yes

**Response:**

**Thanks for your comment and approval.**

4. Have the authors made all data underlying the findings in their manuscript fully available?
The [PLOS Data policy](http://www.plosone.org/static/policies.action#sharing) requires authors to make all data underlying the findings described in their manuscript fully available without restriction, with rare exception (please refer to the Data Availability Statement in the manuscript PDF file). The data should be provided as part of the manuscript or its supporting information, or deposited to a public repository. For example, in addition to summary statistics, the data points behind means, medians and variance measures should be available. If there are restrictions on publicly sharing data—e.g. participant privacy or use of data from a third party—those must be specified.

Reviewer #1: Yes

**Response:**

**Thanks for your comment and approval.**

5. Is the manuscript presented in an intelligible fashion and written in standard English?
PLOS ONE does not copyedit accepted manuscripts, so the language in submitted articles must be clear, correct, and unambiguous. Any typographical or grammatical errors should be corrected at revision, so please note any specific errors here.

Reviewer #1: Yes

**Response:**

**Thanks for your comment and approval.**

6. Review Comments to the Author
Please use the space provided to explain your answers to the questions above. You may also include additional comments for the author, including concerns about dual publication, research ethics, or publication ethics. (Please upload your review as an attachment if it exceeds 20,000 characters)

Reviewer #1: Author answered all the questions raised by me while reviewing the manuscript, satisfactorily. No further comments on the manuscript.

**Response:**

**Thanks for your comment and approval.**

7. PLOS authors have the option to publish the peer review history of their article ([what does this mean?](https://journals.plos.org/plosone/s/editorial-and-peer-review-process#loc-peer-review-history)). If published, this will include your full peer review and any attached files.

If you choose “no”, your identity will remain anonymous but your review may still be made public.

**Do you want your identity to be public for this peer review?** For information about this choice, including consent withdrawal, please see our [Privacy Policy](https://www.plos.org/privacy-policy).

Reviewer #1: No
**Response:**

**Thanks for your comment and approval.**
